# FNS allows efficient event-driven spiking neural network simulations based on a neuron model supporting spike latency

**DOI:** 10.1038/s41598-021-91513-8

**Published:** 2021-06-09

**Authors:** Gianluca Susi, Pilar Garcés, Emanuele Paracone, Alessandro Cristini, Mario Salerno, Fernando Maestú, Ernesto Pereda

**Affiliations:** 1 Laboratory of Cognitive and Computational Neuroscience (Center for Biomedical Technology), Technical University of Madrid & Complutense University of Madrid, Madrid, Spain; 2 Department of Experimental Psychology, Cognitive Processes and Logopedy, Complutense University of Madrid, Madrid, Spain; 3 School of Engineering, University of Rome ‘Tor Vergata’, Rome, Italy; 4 Networking Research Center on Bioengineering, Biomaterials and Nanomedicine (CIBER-BBN), Madrid, Spain; 5 Department of Industrial Engineering & IUNE, University of La Laguna, San Cristóbal de La Laguna, Spain

**Keywords:** Computational biology and bioinformatics, Neuroscience, Mathematics and computing

## Abstract

Neural modelling tools are increasingly employed to describe, explain, and predict the human brain’s behavior. Among them, spiking neural networks (SNNs) make possible the simulation of neural activity at the level of single neurons, but their use is often threatened by the resources needed in terms of processing capabilities and memory. Emerging applications where a low energy burden is required (e.g. implanted neuroprostheses) motivate the exploration of new strategies able to capture the relevant principles of neuronal dynamics in reduced and efficient models. The recent *Leaky Integrate-and-Fire with Latency* (LIFL) spiking neuron model shows some realistic neuronal features and efficiency at the same time, a combination of characteristics that may result appealing for SNN-based brain modelling. In this paper we introduce FNS, the first LIFL-based SNN framework, which combines spiking/synaptic modelling with the event-driven approach, allowing us to define heterogeneous neuron groups and multi-scale connectivity, with delayed connections and plastic synapses. FNS allows multi-thread, precise simulations, integrating a novel parallelization strategy and a mechanism of periodic dumping. We evaluate the performance of FNS in terms of simulation time and used memory, and compare it with those obtained with neuronal models having a similar neurocomputational profile, implemented in NEST, showing that FNS performs better in both scenarios. FNS can be advantageously used to explore the interaction within and between populations of spiking neurons, even for long time-scales and with a limited hardware configuration.

## Introduction

Today’s advanced *magnetic resonance imaging* (MRI)-based techniques allow a thorough estimation of the structural *connectome* (i.e., the map of physical connections in the brain), as well as volume and morphology of single brain areas.

**Figure 1:**
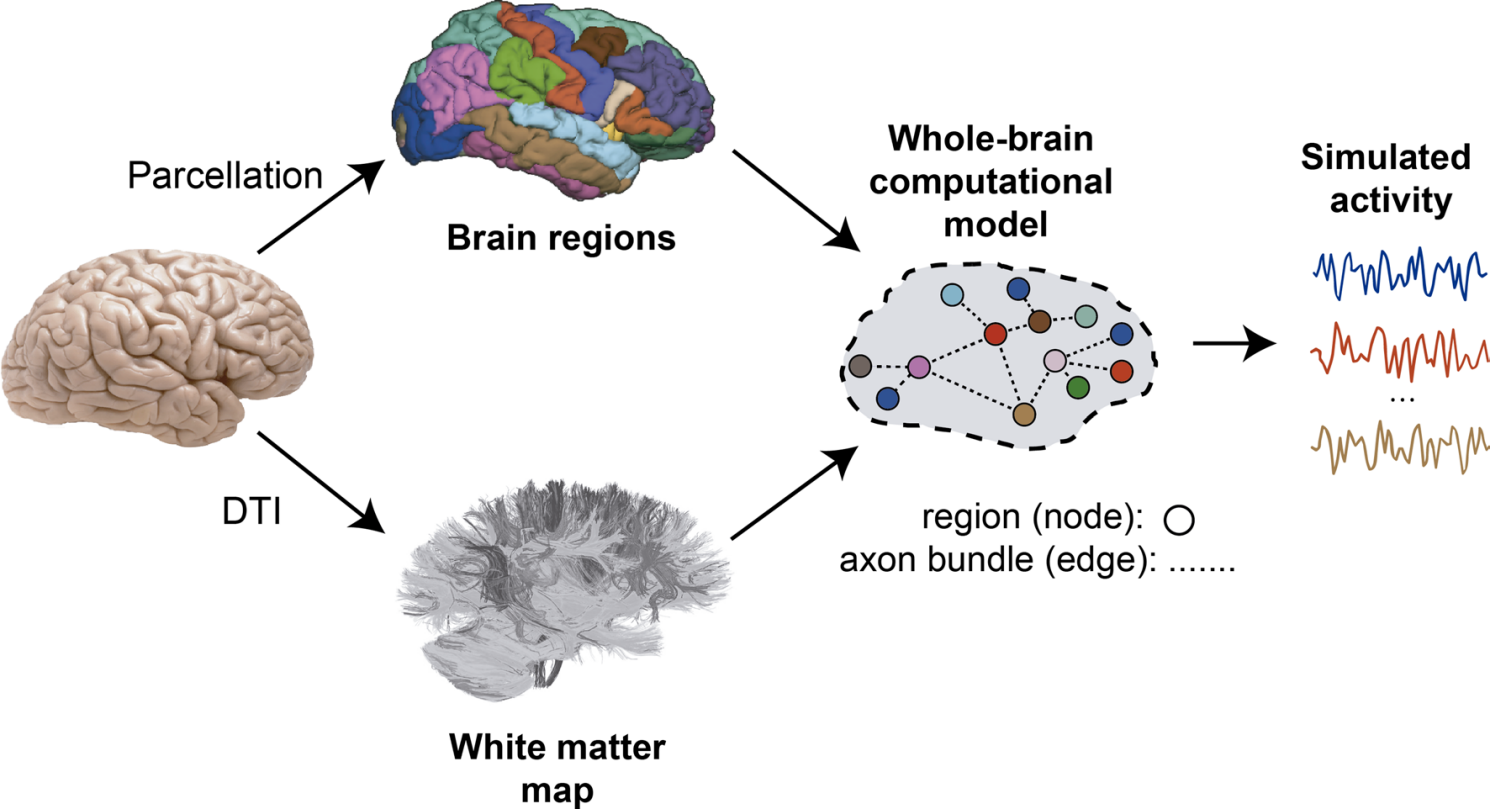
Synthesis of a computational brain model using the graph approach. White matter connections can be extracted by means of DTI. Brains of individual subjects can be coregistered to a parcellation template (*atlas*) in order to assign connections to couples of brain areas. After conferring local dynamics to the nodes of the *structural connectome* obtained, the network activity emerges from the interaction of the component nodes. The number of nodes of the model depends on the template used, and each node can be represented at different levels of abstraction (e.g., ensemble of spiking neurons).

Through the application of graph theory, such data can be employed to synthesize dynamic brain models, which have shown to appropriately reproduce brain oscillations revealed by functional imaging techniques such as *functional* MRI^1,2^, *Magnetoencephalography/Electroencephalography* (M/EEG)^3,4^, *Multi-Unit Activity* (MUA) and *Local Field Potential* (LFP)^5^, providing new information on the brain operation. In such approaches, *nodes* represent surrogates of brain regions (corresponding to *gray matter*), and *edges* represent the long-range connections, along fibre tracts, between them (corresponding to *white matter*), usually estimated using techniques based on *diffusion-weighted MRI* data (like the diffusion tensor imaging, DTI) (Fig. 1).

Simulation studies on brain connectomics revealed that transmission delays introduced by the large-scale connectivity play an essential role in shaping the brain network dynamics, not being, however, the only constraint^4,6^. On the other hand, complementary investigation remarks the substantial role of local dynamics in shaping large-scale functional brain states^7^.

Among the approaches used to reproduce the local activity of single brain regions, *spiking/synaptic models*^3,8, 9^ present a very large number of degrees of freedom, capable of giving rise to highly complex and realistic behaviours on a broad frequency range of the related oscillations^5^. In addition, spiking/synaptic models offer the opportunity to relate to real-brain data transversely (*micro-*, *meso-*, and *macro-scale*, referring to the categorisation of Bohland et al.^10^), as well as to easily implement *spike-timing dependent plasticity* (STDP), which is indispensable in many kinds of computational neuroscience studies. On the other hand, spiking/synaptic-based brain simulations present their criticalities, first of all the fact of being computationally expensive. This often translates to the use of oversimplified spiking neurons thereby reducing the realism of the overall brain model. It motivates a continuous exploration of new avenues for brain modelling based on spiking neural networks (SNNs).

Spiking neuron models are usually described by differential equations and simulated with clock-driven (synchronous) algorithms, by means of proper integration methods (see Brette et al.^11^ for an extensive review). In this way the update is done at every tick of a clock **X**(*t*) $\rightarrow$ **X**$(t+dt)$, and involves all network elements (neurons and possibly synapses). Conversely, in the event-driven (or asynchronous) approach a network element is updated only when it receives or emits a spike. Then, such approach does not envisage a periodic update, neither a check of all network elements, in line with the sparseness of brain-like activity. Nevertheless, the need of an explicit solution for the neuron state between spikes, and the consideration of incoming and outgoing pulses as discrete events, make the event-driven simulation of classic bio-realistic models very challenging. This has stimulated a big interest among the scientific community in developing both realistic and event-driven-compatible spiking neuron models^12–15^, which has led to the development of event-driven based SNN simulators^16,17^, and hybrid *event*/*time-step* based simulation strategies^18–22^. In particular, the *Leaky Integrate-and-Fire with Latency* (LIFL) model is a recent neuron model that can be simulated in event-driven fashion, preserving important computational features at the same time^17,23–26^. LIFL supports relevant neuronal features among which *spike latency*^27–29^, which has been embedded in the model through a mechanism extracted from the *Hodgkin-Huxley* (HH) equations (as described by Salerno and colleagues^14^), and has proved to bring valuable qualities for neural computation^30,31^, as well as beneficial role at the group level as desynchronization^23^ (additional effects of spike latency have been reported by other authors, and summarized in “Methods” section). Then, the LIFL represents an interesting candidate for the event-driven simulation of brain networks. In this work we present FNS (which stands for *Firnet NeuroScience*), a LIFL-based event-driven SNN framework, implemented in *Java* and aimed at exploring the underpinnings of brain network dynamics. FNS allows the user to generate networks of interacting neurons on the basis of a versatile graph-based multi-scale neuroanatomical connectivity scheme, allowing for heterogeneous neuron groups and connections. FNS puts the focus to the reproduction of neuronal activity considering real long-range structural data, even with limited computing resources. FNS uses a novel neuron model, with the possibility to implement diversity at the level of both regions and connections and the option of enabling STDP. In addition to the high customizability of the network, proper input and output modules allow the user to relate model activity to real data. Through the node parameters it is possible to achieve a rich repertoire of intrinsic dynamics, and a set of structural connectivity matrices enables the interaction between the network nodes via specific connection weights, time delays and type of connections.

While a high level of biological detail is necessary for a class of neural modeling studies, such an approach is not always the right key to interpret emergent complex neural dynamics. There are numerous neuroscience studies in which the understanding of neural mechanisms can be facilitated by the use of reduced models. For example, regarding spiking neurons it has been shown that a rich repertoire of states can be obtained even with a few computational ingredients^32^ (see, e.g., Brochini and colleagues^33^ for criticality and phase transitions, and Bhowmik and colleagues^34^ for metastability and interband frequency modulation). In this direction, with FNS we do not want to propose an alternative to today’s detailed simulation softwares, but rather a compact and efficient tool to explore the interaction within and between neuronal populations, even in a simplified manner. In short, FNS aims to facilitate the study of the network dynamics with regards to single neuron neurocomputational features and properties of long-range connections. FNS gives the possibility both to import DTI-derived structural connectivity matrices, and to design custom networks. In addition, a mechanism of periodic dumping and memory management allows the user to face simulations of long-term behavior also with limited hardware. The latter is an important aspect if we want to study phenomena that stretch different time-scales such as STDP-related modifications^35^, criticality^36^ or metastability^37^ in large-scale connectivity models.

This formula seems to allow an interesting trade-off between carrying out simulations that capture both neuron behaviors and macro-scale dynamics, and being able to grasp the contribution of the different computational features. In this regard, the LIFL allows the user to activate/deactivate independently each single feature, to study their effect, either individually or combined, on the network dynamics.

In “Results” section, we evaluate the performance of FNS in terms of simulation time and used memory, making a comparison with the software NEST, considering neuron models similar to the LIFL.

In “Discussion” section, we summarize our work and envisage how to improve FNS in future works.

In “Methods” section, we describe the neurobiological principles and mathematical models underlying FNS, the possibilities that the framework offers for the synthesis of custom models and the design of specific simulations. The salient technical aspects of the simulation framework (e.g., design principles, event-driven implementation and parallelization strategy) are reported in the Appendices (Supplementary information).

In this manuscript, a single neuron is designated with *n*; an axonal connection between two neurons, with *e*; a neuron population (corresponding to a region or subregion in real case), with *N*, and called *network node*; the complete set of connections between two nodes (corresponding to fibre tracts of the real case) with *E*, and called *network edge*.

The software can be freely downloaded at the official FNS website: http://www.fnsneuralsimulator.org.

On the website, a user guide (including a short description of how to install and run it) and some network models are also provided with the software.

## Results

### Simulation examples and performance evaluation

We evaluated the performance of FNS in terms of simulation time and memory usage considering two different scenarios. First, we analyze the scaling behavior with respect to the network size and to the simulated biological time, considering a single node. Then, we test the effectiveness of the parallelization mechanism through the simulation of 14 nodes interconnected with a *connectome-like* structure. Finally, we compare the behavior of the neuron models considered. Given the dual interest in simulating long timescales and obtaining data for future analysis, we have as prerequisite the storage of the data on disk, to avoid out-of-memory errors. We chose to compare FNS with NEST^21,38^, which is one of the most used simulators today and integrates useful commands to write the simulation output to file, disabling the recording to memory (allowing us to execute a fair comparison between the simulators). In NEST we have considered neuronal models that present neurocomputational profiles similar to that of the LIFL: *IAF_psc_delta* and *AEIF_psc_delta* , i.e., the *leaky integrate and fire with delta synapses* and the *adaptive exponential integrate-and-fire with delta synapses*, respectively. While IAF neuron does not support spike-latency, the AEIF is the simplest model available in NEST with this feature^39^; for this latter, we disabled both subthreshold and spike-triggered adaptation, by initializing $a_{AEIF}=b_{AEIF}=0$. For completeness, we finally compared the LIFL with the *precise-spiking* version of the IAF (i.e., the *IAF_psc_delta_ps*^40^), that is the simplest precise-spiking neuron model available in NEST (i.e., characterized by the fact that the location of an outgoing spike is not grid-constrained and determined analytically). The simulations have been carried out using a laptop equipped with *Intel(R) Core(TM) i7-2670QM* CPU and *8GB* of RAM. We used the following software versions: NEST 2.20 (https://zenodo.org/record/3605514#.YHitjuhLjIV) and FNS 3.3.92 (https://github.com/fnsneuralsimulator/versions/tree/main/FNS_3.3.92). Other simulation details are present in the Appendix F.

**Figure 2:**
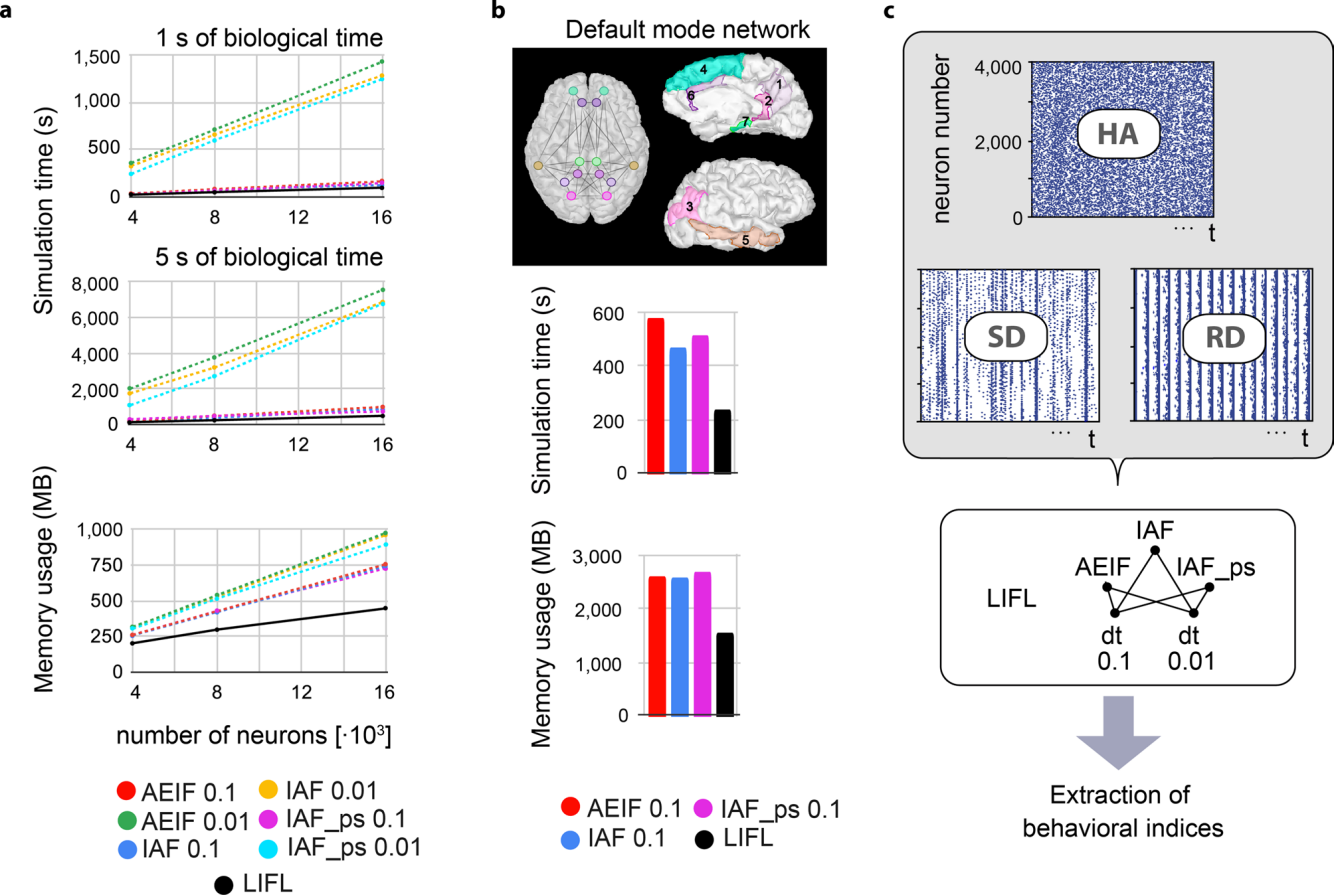
Results from the simulation benchmarks. (**a**) Benchmark A, where the *LIFL* (implemented in FNS) is compared with the neuron models *AEIF with delta synapses* and *IAF with delta synapses* (*grid-based* and *precise-spiking* versions) with resolutions of 0.1 ms and and 0.01 ms (implemented in NEST). Up: simulation time as a function of the size of the network. The simulations have been repeated for 1 s and 5 s of biological time. Each reported value is an average over 5 simulation runs with different randomly generated networks of the same type. Down: used memory as a function of the size of the network. In this case we show only one plot representative of the two cases (1 s and 5 s), since the duration does not affect the memory usage significantly. (**b**) Benchmark B. Comparison of simulation time and memory usage between the *LIFL* (implemented in FNS) and the neuron models of Benchmark A with lower resolutions. A scheme of the populations considered for the DMN is reported, with related localization in the right emisfere: 1—precuneus; 2—isthmus cingulate; 3—inferior parietal; 4—superior frontal; 5—middle temporal; 6—anterior cingulate; 7—(para/) hippocampal. (**c**) Scheme of the simulation battery carried out for the behavioral analysis.

#### Benchmark A: one randomly intra-connected node

As a first neural network example we simulated the *benchmark 4* network model of Brette et al.^11^, which is a random connectivity network with *voltage jump* synapses (i.e., the spikes consist in Dirac pulses). The network is composed of 4000 neurons with a connection probability of $2\%$, and arranged in 2 pools, one excitatory and one inhibitory, forming $80\%$ and $20\%$ of the neurons, respectively. The neurons are characterized by a decay constant of 20 ms and a refractory period of 5 ms. Firing threshold is fixed to $-50 \; \text{mV}$ and reset potential to $-60\; \text{mV}$, and their initial membrane potential is randomly chosen between these two levels of potential. Neuron interactions are permitted by delta synapses, such that each excitatory event causes an instantaneous increase of 0.25 mV on the membrane potential of the target neuron, whereas an inhibitory event causes a decrease of 2.25 mV. For FNS such set of parameters has been adapted to the LIFL neuron (see Appendix F).

We feed the network with a set of 4000 external Poisson processes with mean frequency of 5 Hz, each one connected to 10 randomly chosen neurons, obtaining a mean firing rate activity of $\sim 10\;\text{Hz}$ from each neuron. NEST simulations have been executed for different values of time resolution (i.e., 0.1 ms and 0.01 ms), while in FNS the time is defined as a floating-point variable. In addition to the network already described, we repeated the test using networks of 8,000 and 16,000 neurons. To obtain the same mean firing rate in all the considered networks, we preserved the same balance between excitatory and inhibitory neurons and scaled the number of inputs and connections accordingly (see Table 1).

As for simulation times, LIFL showed significantly better performance compared to IAF, IAF_ps and AEIF with time resolution 0.01 ms, and slightly better performances than the IAF, IAF_ps and AEIF with time resolution 0.1 ms. In terms of RAM usage, LIFL has shown significantly higher performance than the other models/implementations considered. Results are summarized in Fig. 2a.

**Table 1:** Summary of the network parameters for benchmarks A and B.

Benchmark ID	Number of external inputs	Number of internal neurons	Number of input connections	Number of intra-node excitatory connections	Number of intra-node inhibitory connections	Number of inter-node (excitatory) connections
$A_{1}$	4k	4k	40k	256k	64k	–
$A_{2}$	8k	8k	80k	512k	128k	–
$A_{3}$	16k	16k	160k	1024k	256k	–
*B*	56k	56k	560k	3584k	896k	$\sim 100k$

#### Benchmark B: 14 interconnected nodes

Here we evaluated the performance of FNS in a multi-threading scenario, considering 14 nodes of the type described in the benchmark $A_1$ (4*k* neurons), connected using data of the human structural connectome. In particular a well-known brain subnetwork has been considered, the *Default Mode Network* (DMN). The use of real structural data here is only a pretext to make nodes interact with different strengths and delays (our aim here is not to model the real interactions that take place in the brain). We modeled the connectivity and spatial organization of the DMN using DTI data extracted from real subjects, considering the mean lengths and the number of tracts that connect the brain regions of which the DMN is composed. The 14 neuron populations have been placed as vertices of the synthetic DMN and interconnected through excitatory-to-excitatory inter-node connections; to ensure a considerable interaction between the network nodes we have uniformly raised the inter-node weights of the network edges until we obtained a mean firing rate activity of $\sim 12\;\text{Hz}$ considering the neurons of the overall network.

We considered the same neuron models used in the Benchmark A. For NEST, we considered as time resolution only the value 0.1 ms, assumed that with 0.01 ms, lower performance in terms of memory and simulation times are expected. Network parameters are summarized in Table 1.

LIFL outperformed IAF, IAF_ps and AEIF implemented in NEST, both in terms of simulation times and RAM usage. Results are shown in Fig. 2b.

#### Behavioral analysis and comparison

Finally, we carried out a battery of simulations to quantify the behavioral differences between the models under consideration, analyzing the spike patterns resulting from three single-node configurations in specific working regimes: *homogeneous activity* (HA), *sporadic discharges* (SD), and *regular discharges* (RD). To obtain the HA regime, we simply considered a node organized as in benchmark $A_1$ (4000 neurons, mean firing rate of $\sim 10\;\text{Hz}$), where the activity is homogeneous. To obtain the SD regime, we made the module fully connected and reduced the input strength to reach an average firing rate of $\sim 1\;\text{Hz}$, characterized by sparse activity with occasional synchronous neuronal discharges. To obtain the RD regime, starting from $A_1$ we doubled the connection probability and set excitatory and inhibitory weights to an equal, opposite value, achieving frequent and regular synchronous neuronal discharges; the firing rate has been subsequently adjusted to a value of $\sim 50\;\text{Hz}$ through the variation of the input strength. To extract a measure of inter-model similarity, for each network configuration and neuron model we simulated a set of 30 trials (of 1*s*, except for SD, for which we simulated trials of 4*s* to take in account the lower firing rate), considering 0.01*ms* time resolution for the time-driven neuron implementations. Before each trial we re-synthesized the node and initialized the neurons’ membrane potentials to random values, to avoid effects related to peculiar wiring configurations or initial conditions. Finally we evaluated the two following indices from the spike activity produced by the combinations of network configuration/neuron model: (1) the amplitude of the highest peak of the PST-histogram considering bins of 1 ms ($max_{PSTH}$) , and (2) the count of high-synchrony peaks of the PST-histogram considering a threshold of $c_{th} = 100 \, spikes$ ($c_{hi-PSTH}$). To obtain a measure of information degradation deriving from missed spikes, we repeated the set of simulations for 0.1*ms* time resolution, and evaluated the firing rate decrease (*FRd*), that is, the percentage reduction of the average firing rate as a consequence of time-step increase (from $dt=0.01$ to $dt=0.1$). A scheme of the comparison process is given in Fig.2c, and the results are summarized in Table 2.

Considering the three regimes on the whole, AEIF and LIFL are the models whose behavior is most similar, presumably reflecting the affinity of their neurocomputational profile. Specifically, in the SD example they present a similar value of $c_{hi-PSTH}$ and $max_{PSTH}$, while IAF e IAF_ps models behave differently, even from each other. As for the artifactual effects deriving from the variation of time sampling, $FRd_{0.01 \rightarrow 0.1}$ identifies that IAF presents a significant decrease of average firing rate when switching simulation resolution from $dt=0.01$ to $dt=0.1$ in the SD regime. Expectedly, the IAF_ps proved to be robust to this phenomenon, since it is specialized to perform integration with continuous spike times in discrete-time simulations^19^; although for the AEIF this phenomenon was not substantial for the considered regimes and time resolutions, a complementary set of simulations concerning the same regime highlighted discrete spike losses at different resolution ratios ($FRd_{0.001 \rightarrow 0.01}=2.02$; $FRd_{0.001 \rightarrow 1}=9.03$). Considering that in FNS the events are integrated in a continuous-time domain, this parasitic effect is not possible for the LIFL (for this reason the related resolution reduction tests are not contemplated by the table). For completeness, considering each combination of network configuration/neuron model we compared the values of $max_{PSTH}$ and $c_{hi-PSTH}$ related to the simulation sets performed with the two different resolutions, without observing substantial differences.

In conclusion, regarding the three explored regimes, AIF_ps is robust to loss of spikes compared to AIF, but neither supports latency. The AEIF model is the most similar to the LIFL. The latter has proven versatile as it supports latency like the AEIF, and the event-driven implementation makes it devoid of parasitic effects related to time-resolution. Considering also the performance benefits showed in the case of interconnected nodes (especially the reduced memory consumption in benchmark A; both memory consumption and simulation times in benchmark B), LIFL can be advantageously used in different simulation scenarios.

**Table 2:** Behavioral measures obtained with HA, SD, RD regimes.

$Network \,\, configuration$	$Neuron \,\, model$	$max_{PSTH} , dt=0.01$	$c_{hi-PSTH} , dt=0.01$	$FRd_{0.01 \rightarrow 0.1}$
*HA* (F.R.$\approx 10\;\text{Hz}$)	AEIF	66.99 (1.76)	0 (0)	n.r.
	IAF	65.34 (2.66)	0 (0)	n.r.
	IAF_ps	63.32 (0.72)	0 (0)	n.r.
	LIFL	68.11 (1.94)	0 (0)	–
*SD* (F.R.$\approx 1 \;\text{Hz}$)	AEIF	160.49 (95.15)	0.88 (0.99)	n.r.
	IAF	243.31 (70.4)	6.00 (2.56)	$13.53\%$
	IAF_ps	97.25(81.98)	0.25 (0.7)	n.r.
	LIFL	171.07 (114)	1.12 (0.81)	–
*RD* (F.R.$\approx 50 \;\text{Hz}$)	AEIF	2925.5 (61.21)	48.85 (0.73)	n.r.
	IAF	2956.48 (27.3)	49.4 (0.48)	n.r.
	IAF_ps	2803.65 (11.255)	49.8 (0.51)	n.r.
	LIFL	3020 (25.50)	50 (0.41)	–

Both mean and standard deviation are reported for $max_{PSTH}$ and $c_{hi-PSTH}$. The index *FRd* was not computed for the LIFL model since this simulation did not involve time steps; for the other models, only values $\ge 2$ have been considered relevant and then reported (*n*.*r*. instead).

## Discussion

Dynamic models of brain networks can help us to understand the fundamental mechanisms that underpin neural processes, and to relate these processes to neural data. Among the different existing approaches, SNN-based brain simulators allow the user to perform a structure-function mapping at the level of single neurons/synapses, offering a multi-level perspective of brain dynamics.

Here we present FNS, the first neural simulation framework based on the LIFL model, which combines spiking/synaptic neural modelling with the event-driven simulation technique, able to support real neuroanatomical schemes. FNS allows us to generate models with heterogeneous regions and fibre tracts (initializable on the basis of real structural data), and synaptic plasticity; in addition, it enables the introduction of various types of stimuli and the extraction of outputs at different network stages, depending on the kind of activity to be reproduced.

FNS is not an alternative to today’s detailed simulation softwares, but rather a compact and efficient tool to simulate brain networks, constrained by real structural large-scale brain connectivity schemes with the nodes’ intrinsic dynamics originated by spiking neuron-based populations. The framework is based on previous studies which emphasize two basic findings:

- the importance of long-range delays in sustaining interaction patterns between areas of resting-state networks. Specifically, the inclusion of DTI-derived long-range connectivity data is able to contribute notably in shaping the network dynamics, resulting in an increase of the fit among the model and the real case^3, 4^.
- the importance of local dynamics in shaping large-scale functional brain states^7^ and, in particular, the need to have spiking models to relate specific neuronal features to brain network dynamics. FNS allows the user to easily inspect the contribution of some neural features on the network operation, investigating their impact on the spectral properties and implication in (within- and cross- frequency) functional coupling. Among these, it is important to mention the latency, which has been shown to have important implications at the level of neuronal assembly, fostering higher frequencies^25^, and conferring robustness to noise^41^, as well as desynchronizing^14,23^ and stabilization properties^29^ .

FNS would provide the scientific community with a tool to easy understand how these two aspects are able to influence the signal characteristics and the functional connectivity profile in networks of interconnected neuron populations.

FNS gives the possibility to both create custom networks, and import large-scale connectivity structures directly from DTI-derived matrices; through two output files (representing spiking and postsynaptic activity) the resulting simulated signal can be extracted to evaluate the matching with the related real functional data.

Network models built on the anatomical structure enable use-cases of practical clinical interest, e.g., to interpret the effect of changes in the large scale network structure associated to neurodegenerative diseases^42^$$^?$$. Importantly, FNS allows the study of long-term behavior of neural networks, a task that is computationally challenging even for a small number of nodes^35^. In facts, on one hand, the simulation has to evolve over minutes of biological time to capture the timescales of long-term effects such as that of STDP; on the other hand, the simulation has to be sufficiently precise, to capture modifications of weights and internal states as well as time differences that characterize that processes. In FNS, the event-driven technique guarantees high temporal precision, and the implemented memory dump strategy ensures that the simulated biological time does not reflect in an increase in the RAM usage. It gives the possibility to perform long and precise LIFL-based simulations, even with limited hardware setup. As for the “Achille’s heels” of FNS, one of these could be represented by the minimum inter-node delay of the anatomical model: due to the parallelization strategy implemented, the more the minimum internode delay approaches zero, the more the synchronization steps will intensify, resulting in a worsening of the FNS performance. Another critical aspect, inherited by the event-driven method, is represented by simulations with heavy interaction between the coupled network nodes, since the high number of events could slow down the execution^16^. Nevertheless, the usage scenario of FNS makes these criticalities not an obstacle: connectome-like structures usually foresee delays between populations large enough not to pose a threat to FNS execution (except for fine-grained parcellations, less used in this kind of studies). With regards to the second point, heavy interaction is usually an indication of a bad design of the network, since a weak coupling between the brain network regions is normally supposed^9,43^.

With regards to the tests we carried out, and with reference to the neuron models that have been tested, FNS has proven to be versatile and advantageous both in terms of memory and simulation times. In benchmarks A and B, FNS reported even better performances than a model without latency and worse precision implemented in NEST. Moreover, it has to be noted that the possibility to manually set low-level simulation parameters (i.e., *serialization buffer* and *Java heap size*, see Appendix E) gives us the the possibility to adjust the balance between memory usage and simulation time on the basis of the available computing resources.

**Figure 3:**
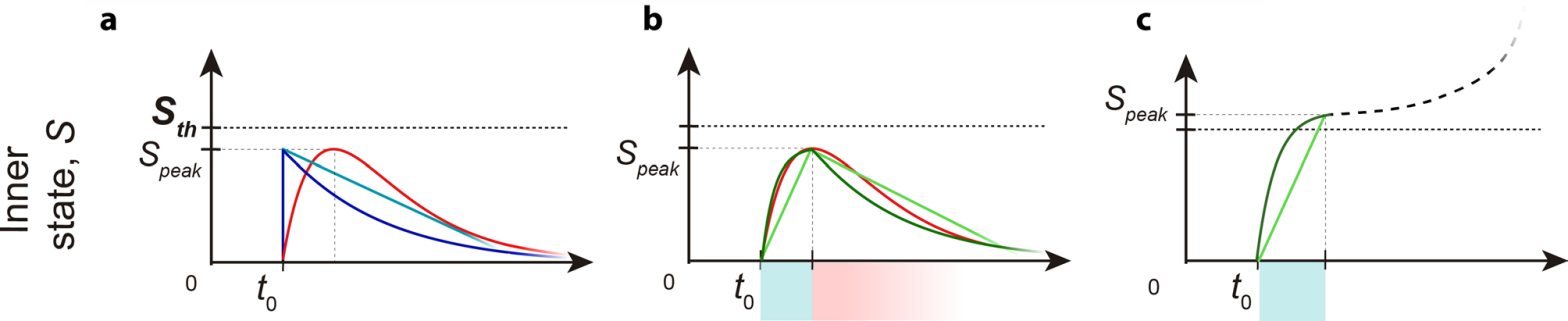
Time course of the neuron’s internal state reached with a single input spike at $t=t_0$ . The alpha shape (red) is compared with (**a**) delta synapses: exponential and linear type (blue and light blue respectively); (**b**) RDI synapses: exp-exp and lin-lin type (green and light green, respectively). In (**c**) we show the internal state evolution in case of threshold crossing considering RDI synapses. The rise and decay phases of RDI approach are indicated through cyan and pink highlights, respectively.

Among the future developments of FNS, we envisage to develop an user-friendly interface and to improve the compatibility with existent *functional-connectivity* estimation tools (e.g., *Hermes*^44^). Then, we have in mind to enrich FNS with new neurocomputational features, both for neurons (e.g., mixed mode and adaptation) and synapses. Regarding the latter, diverse models have been developed to approximate experimentally observed conductance changes (e.g., alpha function^45,46^ and difference of two exponentials^47,48^), which unfortunately are not suitable for event-driven implementations, at least in their original form. A possible strategy for our scenario is to consider the effect of non-instantaneous rise of conductances directly in the neuron’s inner state variable *S*, using piecewise-defined functions. Such mechanism would introduce a rise phase (which takes in account the non-delta behaviour of the synapse), combined with a shift of the starting point of the following phase (i.e., underthreshold decay, or depolarization if the spiking threshold is reached), allowing even to set the time constants of rise and decay independently (a key feature for modeling certain types of synapses^48^), and/or to use different functions (i.e., exponential-exponential, or combined linear-exponential). We show in Fig. 3 a scheme of this mechanism, which we call *rise and decay intervals* (RDI) and in Appendix G the modifications to be done to the current algorithm to embed this feature. The RDI approach is easily implementable in event-driven and would introduce a negligible computational cost because the rise function will be computed only if new contributions actually arrive during the rising phase, otherwise the computation will remain basically unchanged.

Finally, we plan to develop an alternative version of the software characterized by a lower precision of the internal variables, to achieve a further increase in performance (at the expenses of a little amount of introduced error). The current version of FNS is written in Java$(R)$. The software is open-source and published under a free license, permitting modification and redistribution under the terms of the GNU General Public License v.3. The reader can find the software package and technical documentation on the FNS website http://www.fnsneuralsimulator.org.

## Methods

### From neurobiology to mathematical models

Recent works highlight that bioplausibility and diversity characterize the human brain at all scales, and are central aspects to be taken into account to obtain realistic dynamics in brain models, both at intra-region^49,50^ and among-region^8,51–53^ levels.

In this section we present mathematical models used in FNS, aimed at guaranteeing the possibility to take into account such aspects while at the same time focusing on ease of use.

#### LIFL neuron model

Altough the classic LIF model is very fast to simulate, it has been regarded as unrealistically simple, thereby incapable of reproducing the dynamics exhibited by cortical neurons^54^. FNS is based on the LIFL, that besides being computationally simple it is also able to support a greater number of neuronal features than the LIF.

##### A brief introduction to the spike latency neuro-computational feature

 The *spike latency* is the delay exhibited by a neuron in response to a depolarization. It prevents the immediate spike generation and depends on the strength of the input signal^28^. Considering pulses as input, it is the membrane potential-dependent delay time between the overcoming of the “threshold” potential and the actual spike generation. It is an important neurocomputational feature because it extends the neuron computation capabilities over the “threshold”, giving rise to a range of new behaviors. Spike latency is ubiquitous in the nervous system, including the auditory, visual, and somatosensory systems^55,56^.

From a computational point of view it provides a spike-timing mechanism to encode the strength of the input^41^ conferring many coding/decoding capabilities to the network^24,57,58^, whereas, from a statistical point of view, it results in a desynchronizing effect^14,23^, fostering the emergence of higher frequencies^25^ and providing robustness to noise to the network^41^. Interestingly, in the presence of plasticity, spike latency has proven to play an important role in stabilizing and extending polychronous groups, even able to explain unusual results in the dynamic of the weights^29^. Taken together, these findings point out that its inclusion in neuronal models is crucial if the goal is to investigate biologically plausible behaviors emerging from neuron assemblies. Spike latency has already been introduced in some variants of the LIF, as *QIF*^59^ and *EIF*^60^. In LIFL, spike latency is embedded with a mechanism extracted from the realistic HH model^14^, both simple and suitable to the event-driven simulation strategy. LIFL is characterized by a simple and modular mathematical form, so that its neurocomputational features, as the spike latency, can be independently switched on/off, allowing to study their effect on the network dynamics in a single or combined way.

##### LIFL operation

 The LIFL neuron model is characterized by a real non-negative quantity *S* (the *inner state*, corresponding to the membrane potential of the biological neuron), which ranges from 0 (corresponding to the resting potential of the biological neuron) to $S_{max}$ (*maximum state*), a value much greater than one, at most $\infty$. Simple Dirac delta functions (representing the action potentials) are supposed to be exchanged between network’s neurons, in form of *pulse* trains. The model is able to operate in two different modes: *passive mode* when $S<S_{th}$, and *active mode* when $S\ge S_{th}$, where $S_{th}$ is the *state threshold*, a value slightly greater than 1 which corresponds to the threshold potential of the biological neuron. In passive mode, *S* is affected by a decay, whereas the active mode is characterized by a spontaneous growth of *S*. Assuming that neuron $n_j$ (i.e., the *post-synaptic neuron*) is receiving a pulse from neuron $n_i$ (i.e., the *pre-synaptic neuron*), its inner state is updated through one of the following equations, depending on whether $n_j$ was in passive or in active mode, respectively:

1a $$S_{j} = S_{{p_{j} }} + A_{i} \cdot W_{{i,j}} - T_{l} ,\;\;\;{\text{for}}\;\;0 \le S_{{p_{j} }} < S_{{th}}$$

1b $$S_{j} = S_{{p_{j} }} + A_{i} \cdot W_{{i,j}} + T_{r} ,\;\;\;{\text{for}}\;\;S_{{th}} \le S_{{p_{j} }} < S_{{\max }}$$

$S_{p\;_{j}}$ represents the post-synaptic neuron’s *previous state*, i.e., the inner state immediately before the new pulse arrives. $A_{_{i}}$ represents the *pre-synaptic amplitude*, which is related to the pre-synaptic neuron, and can be positive or negative depending on whether the neuron sends excitatory or inhibitory connections, respectively.

$W_{_{i,j}}$ represents the *post-synaptic weight* (corresponding to the conductance of the real case); if this quantity is equal to 0, the related connection is not present. The product $A_{i}\cdot W_{i,j}$ globally represents the amplitude of the pulse arriving to the post-synaptic neuron $n_j$ (i.e., the *synaptic pulse*) from the pre-synaptic neuron $n_i$. In this paper, *w* or $\omega$ will be used instead of W, depending on whether the connection is intra- or inter- node, respectively.

$T_l$ (the *leakage term*) takes into account the behaviour of *S* during two consecutive input pulses in passive mode. The user is allowed to select among *linear* or *exponential* underthreshold decays characterized by the *decay parameter*, as explained in the Appendix A. For each node, such parameter can be set with different values for excitatory and inhibitory connections (i.e., $D_{exc}$ and $D_{inh}$) in order to model different synapse types.

$T_r$ (the *rise term*) takes into account the overthreshold growth acting upon *S* during two consecutive input pulses in active mode. Specifically, once the neuron’s inner state crosses the threshold, the neuron is ready to produce a spike. The emission is not instantaneous, but it occurs after a continuous-time delay corresponding to the spike latency of the biological neuron, that we call *time-to-fire* and indicate with $t_f$ in our model. This quantity can be affected by further inputs, making the neuron sensitive to changes in the network spiking activity for a certain time window, until the actual spike generation. *S* and $t_f$ are related through the following bijective relationship, called the *firing equation*:

2 $$\begin{aligned} t_f ={\frac{a}{(S-1)}}-b \; \end{aligned}$$

where $a,b \ge 0$. Such rectangular hyperbola has been obtained through the simulation of a membrane patch stimulated by brief current pulses (i.e., 0.01 *ms* of duration), solving the HH equations^61^ in *NEURON* environment^62^, as described in^14^. Then, if the inner state of a neuron is known, the related $t_{f}$ can be exactly calculated by means of Eq. (2). As introduced in , this nonlinear trend has been observed in most cortical neurons^28^; similar behaviors have been also found by other authors, such as^55^ and^56^, using DC inputs. Conversely to previous versions of LIFL^17,30^, constants *a* and *b* have been introduced in order to make the model able to encompass the latency curves of a greater number of neuron types; in particular, *a* allows us to distance/approach the hyperbola to its centre, while *b* allows us to define a $S_{max}$, conferring a bio-physical meaning to the inner state in active mode (note that if $b=0$, then $S_{max}=\infty$; nevertheless, the neuron will continue to show the spike latency feature).

The $S_{th}$ can be equivalently written as:

3 $$\begin{aligned} S_{th} = 1+c \; \end{aligned}$$

where *c* is a positive value called *threshold constant*, that fixes a bound for the maximum $t_{f}$. According to Eq. (3), when $S = S_{th}$, the $t_{f}$ is maximum, and equal to:

4 $$\begin{aligned} t_{f,max} = a/c - b \end{aligned}$$

where $t_{f,max}$ represents the upper bound of the $t_f$. As mentioned above, the latter consideration is crucial in order to have a finite maximum spike latency as in biological neurons^27^. From the last equation, we obtain the restriction $c<a/b$.

**Figure 4:**
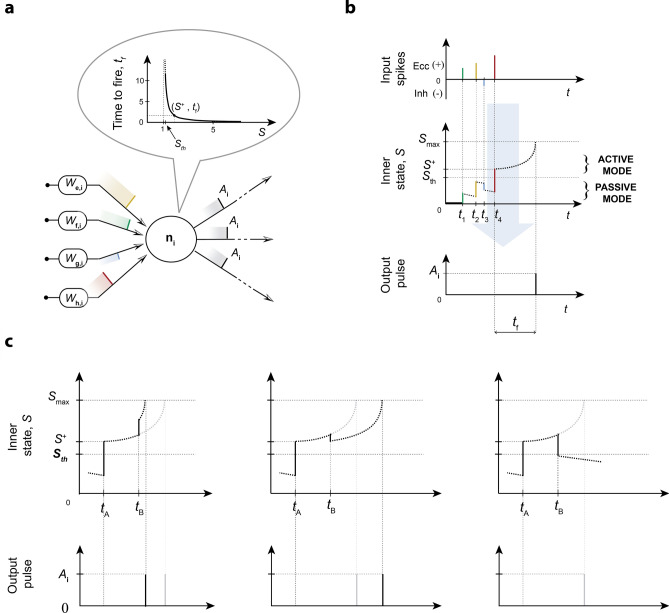
Neural summation and spike generation in a LIFL neuron. (**a**) Input/output process scheme, with firing equation curve ($a=1$, $b=0$, $c=0$^+^). (**b**) Temporal diagram of LIFL operation (basic configuration). Excitatory (inhibitory) inputs cause an instantaneous increase (decrease) of the inner state. When *S* exceeds $S_{th}$ the neuron is ready to spike; due to the latency effect, the spike generation is not instantaneous but it occurs after $t_{f}$. (**c**) Effect of the arrival of further inputs when the neuron is overthreshold. An excitatory synaptic pulse is able to (left) anticipate the spike generation (post-trigger anticipation); an inhibitory synaptic pulse is able to (center) delay the spike generation (*post-trigger postponement*), or (right) to cancel the spike generation (*post-trigger inhibition*). The state evolution in the simple case of no further inputs is reported in grey.

As described in Appendix B, using Eq. (2), it is possible to obtain $T_r$, as follows:

5 $$\begin{aligned} T_r =\frac{(S_{p}-1)^{2} \Delta t}{a-(S_{p}-1)\Delta t} \; \end{aligned}$$

in which $S_{p}$ represents the previous state, whereas $\Delta t$ is the temporal distance between two consecutive incoming pre-synaptic spikes. The Eq. (5) allows us to determine the inner state of a neuron at the time that it receives further inputs during the $t_f$ time window. In Fig. 4 are shown both the operation of LIFL and the effect of Eq. (5).

Assuming that an input spike leads the inner state overthreshold at time $t_{A}$, the arrival of a contribution during the latency time (i.e., at time $t_{B}$) results in a new $t_f$. Excitatory (inhibitory) inputs increase (decrease) the inner state of a post-synaptic neuron. Therefore, when a neuron is in active mode, excitatory (inhibitory) inputs decrease (increase) the related $t_f$ (*post-trigger anticipation/postponement* respectively). If the inhibitory effect is as strong as to pull the post-synaptic neuron state under $S_{th}$, its $t_{f}$ will be suppressed and its state will come back to the passive mode (*post-trigger inhibition*)^14,17^.

For a given neuron *j* in active mode, the arrival of new input contributions implies $t_{f}$ updating. Once the $t_{f}$ is reached, the output spike is generated and the inner state is reset. Note that if incoming spikes are such as to bring $S<0$, *S* is automatically set to 0. Differently, if incoming spikes bring $S> S_{max}$, a spike is immediately generated. We emphasize the fact that spike latency enables a mechanism to encode neural information, supported from all the most plausible models. Thus, there is lack of information in models that do not exhibit this relevant property.

Hitherto we have discussed a basic configuration of LIFL, which defines an intrinsically *class 1 excitable*, *integrator* neuron, supporting *tonic spiking* and *spike latency*. Nevertheless, thanks to the simplicity of its mathematical model, it can be enriched with some other neuro-computational features to reproduce different kinds of cortical neurons^28^ by introducing minimal modifications to the model equations, or by adding extrinsic properties at the programming level. This is the case of *refractory period* for which the neuron becomes insensitive, for a period $t_{arp}$, to further incoming spikes after the spike generation, and *tonic bursting* for which the neuron produces a train of $N_{b}$ spikes spaced by an inter-burst interval *IBI*, instead of a single one.

In addition to the spike latency, emerging from the neuron’s equations, in the next section another kind of delay will be introduced, to characterize the long-range connections between neurons belonging to different groups.

#### Connection between 2 neurons

In FNS the network nodes are groups of spiking neurons to represent brain regions. Neurons of the same node interact instantaneously, whereas a settable time delay ($\ge 0$) is present between neurons of different nodes to reflect the remoteness between the regions to which they pertain.

**Figure 5:**
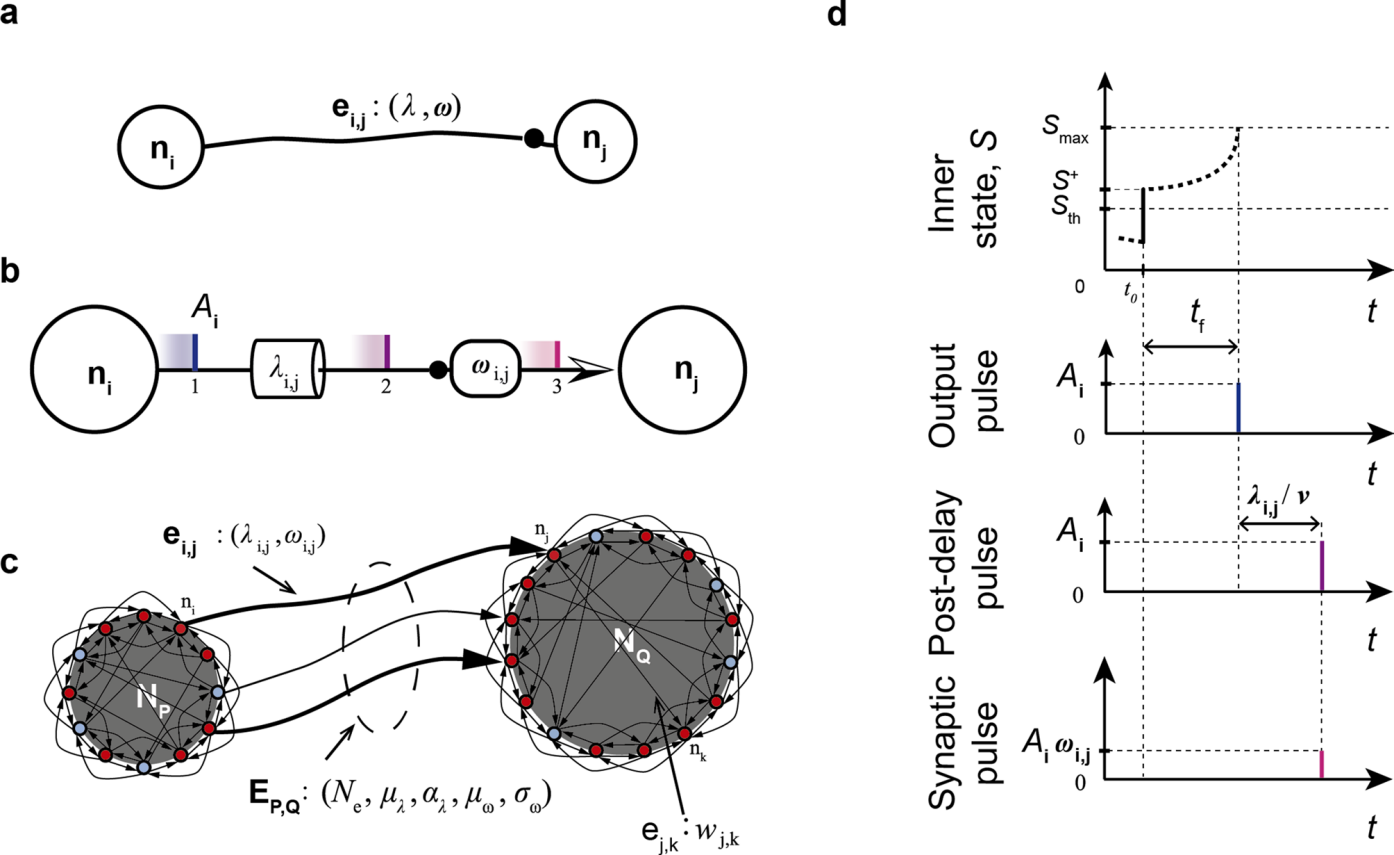
Neuron connection model and pulse transfer. (**a**) Compact representation and (**b**) logical block representation, where the black dot represents synaptic junctions. (**c**) Two nodes connected by an edge. While an intra-node connection is characterized by its weight, an inter-node connection is defined by weight and length; an edge is described by number of axons and related distribution of weights and lengths. (**d**) Inter-node diagram considering an excitatory neuron: $\lambda$ produces a translation of the output pulse along time axis, while $\omega$ acts on the pulse amplitude. *Output pulses* represent the spiking activity, whereas *synaptic pulses* represent the synaptic currents.

A scheme of inter-node neuron connection ($e_{i,j}$) is illustrated in Fig. 5, where $\lambda _{i,j}$ represents the *axonal length* block and $\omega _{i,j}$ represents the *post-synaptic weight* block. Such two link elements (belonging to a directed connection) are able to introduce delay and amplification/attenuation of the passing pulse, respectively. As in^3, 4^ a global propagation speed *v* is set for FNS simulations, so that inter-node connection delays are automatically defined from the axonal lengths, as $\tau _{i,j}=\lambda _{i,j} / v$. Connection delays are important since they allow to take into account the three-dimensionality (i.e., spatial embeddedness) of the real anatomical brain networks.

For the reasons mentioned before, conversely to the inter-node connection (represented as $E_{i,j}$ in Fig. 5), the intra-node connection (represented as $e_{j,k}$ in the same figure) does not provide the axonal length block (although synaptic weight block continues to be defined).

For biological and mathematical reasons, it is desirable to keep the synaptic weights under a certain value, $W_{max}$, a global parameter of the model.

In the following sections we call *firing event* the pulse generation by a pre-synaptic neuron, and *burning event* the pulse delivery to a post-synaptic neuron.

#### From brain regions to graph nodes

FNS allows us to define regions constituted by one or more *nodes* where each node consists of a neuron group with specific properties. In order to reproduce heterogeneous nodes, a Watts-Strogatz based generative procedure is implemented as detailed below, allowing the generation of networks with structure properties of real neuron populations.

The implemented procedure allows us to model intra- and inter-node diversity: number of neurons and connectivity, percentage of inhibitory neurons, distribution of weights and type of neuron; in addition, it is possible to represent a region with more than one node to model intra-region neuronal pools of different connectivity and neuron types. In the extreme case, a group can be composed of a single neuron, e.g., for reproducing small and deterministic motifs. In the following sections we illustrate the procedure used by FNS for the generation of network nodes and the structure of intra- and inter- node connections.

##### Watts-Strogatz-based node generation procedure

 The original Watts-Strogatz procedure is able to generate different types of complex networks (from regular to random), including networks with *small-world* properties (i.e., networks that present large *clustering coefficient* and small *average path length*), that has been demonstrated to reasonably approximate a patch of cortex with its neighborhood (i.e., coupled both to nearby cells within $50{-}100 \; \upmu \text{m}$, and to some others placed millimeters away^63^). In FNS the original Watts-Strogatz procedure is adapted to generate a group including both inhibitory and excitatory, directed, connections^9^. Given the integer *n* (i.e., *number of neurons*), *k* (i.e., *mean degree*), *p* (i.e., *rewiring probability*), and *R* (i.e., *excitatory ratio*), with $0\le p \le 1$ and $n\gg k \gg ln(n)\gg 1$, the model generates a directed graph with *n* vertices and *nk* single connections in the following way:

- a regular ring lattice of *n* spiking neurons is created, of which $R\cdot n$ are able to send excitatory connections and the remaining $(1-R)\cdot n$ are able to send inhibitory connections;
- for each neuron an outgoing connection to the closest *k* neurons is generated (*k*/2 connections for each side, with $k \le n-1$, integer and even);
- for each neuron *i*, every link $e_{i,j}$ with $i<j$, is rewired with probability *p*; rewiring is done by exchanging $e_{i,j}$ and $e_{i,m}$ where *m* is chosen with uniform probability from all possible (excitatory or inhibitory) neurons that avoid self-loops ($m\ne i$) and link duplication. This process is repeated *n* times, each one considering a different neuron.

Note that the parameter *p* allows to interpolate between a regular lattice ($p=0$) and a random graph ($p=1$): as *p* increases, the graph becomes increasingly disordered. For intermediate values of *p* the network presents small-world properties. The parameters *n*, *k*, *p* allow the user to customize the network nodes on the basis of the real anatomy. For example, in the case of simulation of biological networks *n* can be chosen in accord to the volume of the region that is intended to be represented (estimated from a specific subject through volumetry, or extracted from existing *atlases*).

##### Characterization of intra-node connections

 Once connections have been established, weights have to be assigned. Several authors have addressed this problem, setting intra-node weights in different manners. Depending on the specific study, weights have been chosen to have the same, static value^2^, or characterized by a specific distribution^43^, or varying in a certain range by means of plasticity^64^. In order to encompass the most of these possibilities, in FNS a set of Gaussian distributed values can be defined by the user for the initialization of the intra-node post-synaptic weights, for each of the node.

#### From fibre tracts to graph edges

In FSN an *edge* represents a monodirectional set of long-range axons that links a node to another. In the brain, inter-region connections are often characterized by non negligible delays, which are determined by axon length, diameter and myelination degree. FNS allows the user to evaluate the impact of different edge features on the functional properties of the network.

##### Characterization of inter-node connections

 FNS allows the user to set the number of connections $N_e$ and to specify distribution of weights and lengths for each edge of the network. The distribution of edge weights follows a Gaussian function^43^, characterized by the parameters $\mu _{\omega }$ and $\sigma _{\omega }$. Differently, a gamma distribution is implemented for the edge lengths, characterized by mean parameter $\mu _{\lambda }$ and shape parameter $\alpha _{\lambda }$ , since there is probably not a unique prototypical shape for edge delays, as discussed in previous studies^8^. Indeed, this distribution allows the user to explore different shapes, to investigate the impact of different choices on the network activity, to mimic pathological states as the effect of structural inhomogeneity^65^, or spatially-selective conduction speed decrease due to demyelination. FNS supports STDP a well-known type of plasticity mechanism, believed to underlie learning and information storage in the brain, and refine neuronal circuits during brain development^66^. Importantly, studies have shown that STDP varies widely across synapse types and brain regions^67^. Accordingly, in FNS it is possible to specify a different set of STDP parameters for each node, or to apply STDP uniquely for certain nodes. The implementation aspects of STDP are detailed in Appendix C. Finally, for each edge, the user can specify the type of neurons involved as senders and receivers (i.e., excitatory or inhibitory or mixed, to excitatory or inhibitory or mixed), by means of the parameter $t_E$.

#### Input stimuli

Several types of stimuli can be of interest in brain simulation studies. Of these, two prototypical types of stimuli are:

- the noisy fluctuations tipically observed in vivo, which can be modeled by uncorrelated Poisson-distributed spike trains^3, 43, 68^;
- the DC current used by neurophysiologists to test some neuron features^8,28^.

In addition, in many simulation scenarios the possibility of giving arbitrary spike streams (e.g., sequences that mimic sensory-like processed data) can be of interest, in order to test the response of specific brain subnetworks.

In light of these observations, in FNS it is possible to stimulate brain nodes with three different types of inputs: *Poisson-distributed spike train*, *constant spike train*, and *arbitrary spike stream*. The user is allowed to stimulate all or only a part of the network nodes, choosing for each kind of input a customizable number of fictive excitatory *external neurons*, and the characteristics of the required stimuli. An external neuron is permanently associated to one or more neuron of the related node.

##### Poisson-distributed spike train

 This option provides the injection of Poisson-like spike trains, obtained by an exponential distribution, in which the underlying *instantaneous firing rate* $r_P$ is constant over time.

In FNS, a user-defined number of fictive *external neurons* $n_{ext P,k}$ is set for each stimulated node $N_k$. By defining a $t_{start P,k}$ and a $t_{end P,k}$ for the external stimuli, each external neuron can send spikes in a discrete number of instants $(t_{start P,k} - t_{end P,k})/ \delta t_P$. The target neurons receive pulses of amplitude $A_{P,k}$.

Pulses are injected from each external neuron to the neurons belonging to a set of nodes defined by the user, by specifying the following set of parameters for each chosen node $N_k$: $n_{ext P,k}$, $t_{start P,k}$, $t_{end P,k}$, $r_{P,k}$, $\delta t_{P,k}$ and $A_{P,k}$.

##### Constant spike train

 This option provides the injection of emulated DC current stimulation. Note that since we simulate the network by means of an event-driven approach, the *DC* input is not continuous but it is constantly sampled with an adequately small time step, called *interspike interval* and indicated with $int_{\,c}$.

In FNS, a user-defined number of fictive *external neurons* $n_{ext \,c,k}$ is set for each stimulated node $N_k$. Each external neuron can send spikes from time $t_{start \,c,k}$ to $t_{end \,c,k}$, with amplitude $A_{\,c,k}$. Such kind of input is injected from each external neuron to the neurons belonging to a set of nodes defined by the user, by specifying the following set of parameters for each chosen node $N_k$: $n_{ext \,c, k}$,$t_{start \,c,k}$, $t_{end \,c,k}$, $int_{\,c,k}$ and $A_{\,c,k}$.

##### Arbitrary spike stream

 Arbitrary spike streams can be injected to neurons belonging to a set of nodes defined by the user by specifying the following set of parameters for each chosen node $N_k$: the spike *amplitude* $A_{ss,k}$, and a couple ($n_{ss,k}$, $t_{ss,k}$) for each event to be introduced (i.e., *external source number* and related *spike timing*, respectively).

#### Output signals

Depending on the type of contributions we are considering at the network level, i.e., output pulses (corresponding to *action potentials*) or synaptic pulses (corresponding to *post-synaptic currents*), the same network activity gives rise to different signals, due to the presence of connection delays and weights.

In particular, action potentials coincide with the activity emerging from *firing* events, because they take place before the axon, thus they are spatially localized at the emitter node; whereas post-synaptic currents coincide with the post-synaptic activity, because they take place downstream the axon, thus they are spatially localized to the receiver node, and are affected by the shifting effect introduced by (heterogeneous) fibre tract’s delays and post-synaptic weights.

Action potentials are of interest for some studies^8^, whereas post-synaptic currents can be useful for some others (see^3,69^ for LFP and MEG signal reconstruction).

In order to give the user the possibility to recostruct such different types of signals, output module of FNS allows to store both pulse emission and arrival times ($t_F$ and $t_B$), transmitter and receiver neurons ($n_F$ and $n_B$) and related nodes ($N_F$ and $N_B$), as well as amplitude weights ($W_{ev}$) involved in each event occurring during the simulation interval, for some nodes indicated by the user before the simulation starts.

### Structure of the simulation framework and implementation strategies

On the basis of the modelling introduced, here we describe the framework structure and the tools it offers to the user for implementing a custom network, stimulating it, and obtaining the outputs of interest.

**Figure 6:**
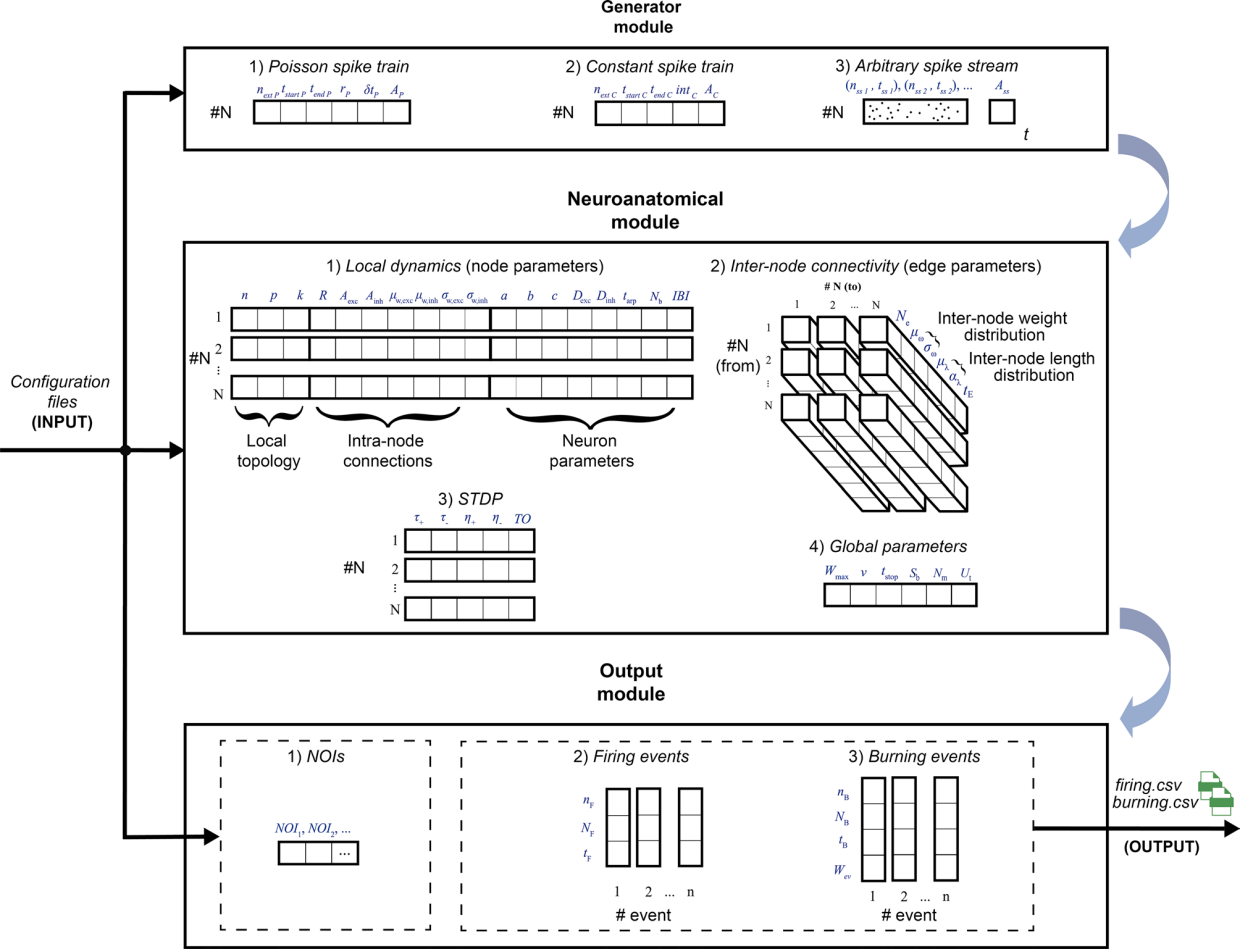
FNS framework overall structure. The reader can find the meaning of the abbreviations in Table 3.

The framework is articulated in three main modules: *Generator module*, *Neuroanatomical module* and *Output module* (see Fig. 6). In order to design a simulation, the user interacts with such modules by means of proper configuration files, which are defined in Table 3.

**Table 3:** Definition of the system parameters.

Module	Components	Name
Generator module	$n_{ext P}$	Number of *Poisson spike train* external neurons
	$t_{start P}$	Poisson input onset
	$t_{end P}$	Poisson input offset
	$r_P$	Firing rate
	$\delta t_P$	Delta
	$A_P$	Poisson input amplitude
	$n_{ext \, c}$	Number of *constant spike train* external neurons
	$t_{start{\,c}}$	Constant input onset
	$t_{end\,c}$	Constant input offset
	$int_{\,c}$	Interspike interval
	$A_{\,c}$	Cnstant input amplitude
	$t_{ss 1}, t_{ss 2}, ...$	*Input stream* spike timings
	$n_{ss 1}, n_{ss 2}, ...$	Related neuron numbers
	$A_{ss}$	Stream input amplitude
Neuroanatomical node module	*n*	Number of neurons
	*p*	Rewiring probability
	*k*	Mean degree
	*R*	Excitatory ratio
	$A_{exc}$	Exc. pre-synaptic amplitude
	$A_{inh}$	Inh. pre-synaptic amplitude
	$\mu _{_w, exc}$	Intra-node exc. post-synaptic weight distr.mean (Gaussian)
	$\mu _{_w, inh}$	Intra-node inh. post-synaptic weight distr.mean (Gaussian)
	$\sigma _{_w, exc}$	Intra-node exc. post-synaptic weight distr.st.dev. (Gaussian)
	$\sigma _{_w, inh}$	Intra-node inh. post-synaptic weight distr.st.dev. (Gaussian)
	*a*	Latency curve center distance
	*b*	Latency curve x-axis intersection
	*c*	Threshold constant
	$D_{exc}$	Decay parameter (excitatory)
	$D_{inh}$	Decay parameter (inhibitory)
	$t_{arp}$	Absolute refractory period
	$N_b$	Burst cardinality
	*IBI*	Inter-burst interval
	$N_{e}$	Number of connections (edge cardinality)
	$\mu _{\omega }$	Inter-node post-synaptic weight distr.mean (Gaussian)
	$\sigma _{\omega }$	Inter-node post-synaptic weight distr.st.dev. (Gaussian)
	$\mu _{\lambda }$	Inter-node length distr.mean (gamma)
	$\alpha _{\lambda }$	Inter-node length distr.shape (gamma)
	$t_{E}$	Inter-node sender-receiver type
	$\tau _{+}$	LTP time constant
	$\tau _{-}$	LTD time constant
	$\eta _{+}$	LTP learning constant
	$\eta _{-}$	LTD learning constant
	*TO*	STDP timeout constant
	$W_{max}$	Maximum weight
	*v*	Global conduction speed
	$t_{stop}$	Simulation stop time
	$S_b$	Serialization buffer
	$N_m$	Neuron model
	$U_t$	Underthreshold type
Output module	$NOI_1, NOI_2, ...$	List of *NOI*s
	$n_{F}$	Pre-synaptic neuron number (if firing event)
	$N_{F}$	Pre-synaptic node number (if firing event)
	$t_F$	Firing event time (if firing event)
	$n_{B}$	Post-synaptic neuron number (if burning event)
	$N_{B}$	Post-synaptic node number (if burning event)
	$t_B$	Pulse arrival time (if burning event)
	$W_{B}$	Synaptic weight (if burning event)

**Figure 7:**
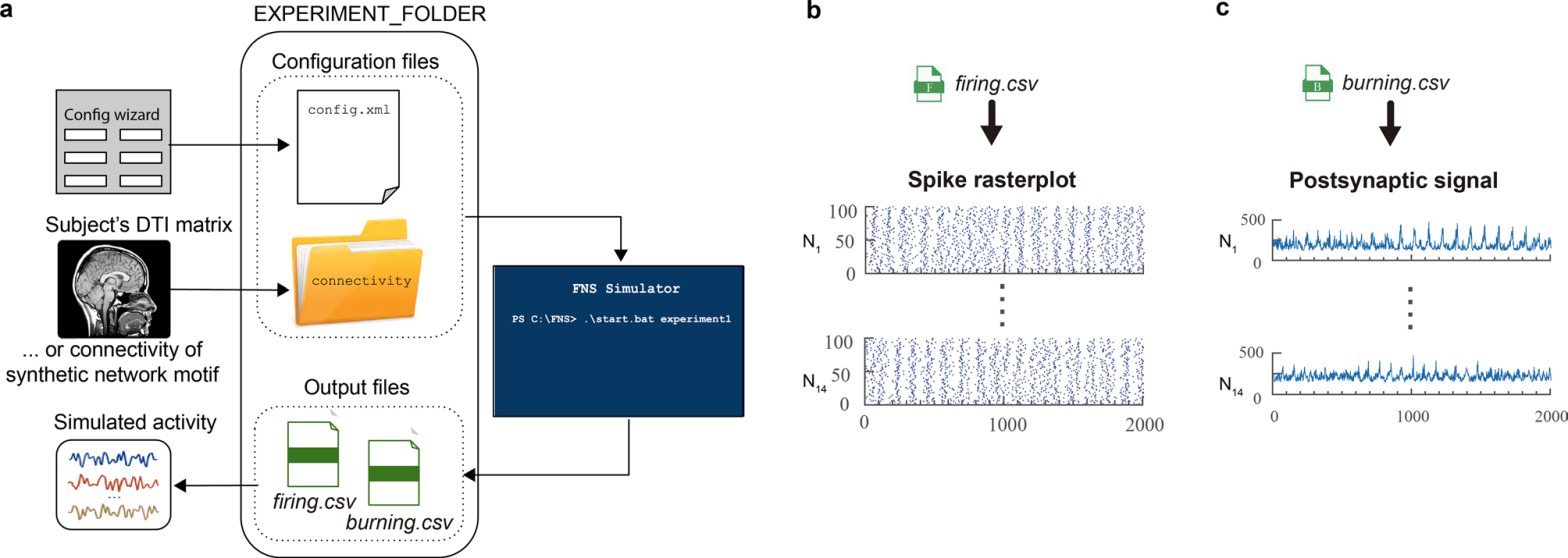
Operation flow of a simulation using FNS. (**a**) the simulated activity is obtained through three steps: (1) FNS is configured through the so-called *config.xml* file (manually or through the dedicated *Config wizard* available on the FNS website), and *connectivity folder*, that together contain the values to setup the *generator module* and *neuroanatomical module*; (2) Simulation through FNS; (3) Reconstruction of the electrophysiological-like signal using the FNS output files (firing.csv and/or burning.csv). (**b**) two seconds of simulated signal are extracted from *firing.CSV* and *burning.CSV* files of a simulation of 14 nodes composed of 100 neurons each. The figures have been obtained throught the dedicated scripts available on the FNS github page: http://github.com/fnsneuralsimulator/FNS-scripts_and_tools.

FNS allows the user to both simulate synthetic network motifs and reproduce real biological networks. A scheme of the simulation steps needed to obtain simulated electrophysiology activity is shown in Fig. 7.

#### Generator module

This module allows the user to inject the desired input to some selected nodes. *Poisson spike train*, *constant spike train* and *arbitrary spike stream* can be combined to send more than a kind of input to the same node simultaneously.

#### Neuroanatomical module

This module allows the user to define the network model: local dynamics, structural parameters, plasticity constants and global parameters. Each node is fully characterized by its *local dynamics* parameters, consisting of *topology parameters*, *intra-node connection parameters* and *neuron parameters*. From the definition of node’s weight distribution, the simulator computes all the single intra-node synaptic weights and stores them in proper data structures (see Appendix D).

Each edge is fully characterized by the *inter-node connectivity* parameters, consisting of *edge cardinality*, *inter-node weight distribution*, *length distribution parameters*, and *sender-receiver type*. From the definition of such parameters the simulator generates the inter-node connections, computes all the related lengths and weights and stores them in proper data structures (see Appendix D). The STDP parameters define the STDP to act on a specific node.

As for the global parameters of the system, $t_{stop}$ specifies the neural activity time we want to simulate in biological time units (*ms*), $N_m$ is a binary variable that allows us to switch among LIFL and LIFL neuron models, and $U_t$ is a binary variable that indicates the underthreshold behaviour to be implemented. The remaining parameters are described along this document.

#### Output module

This module allows the user to choose regions and type of contributions to be recorded during the simulation. Before the simulation starts, the user can specify the list of nodes for which to store all simulation data (i.e., the *nodes of interests* (NOIs)). Data of all firing and burning events in which such NOIs are implicated are collected in a differentiated manner and made available to the user through the two files *firing.CSV* and *burning.CSV*. Such files report exhaustive information on firing events and burning events, for the extraction of simulated electrophysiological signal (firing activity in the first case and postsynaptic activity in the second case, respectively, see Fig. 7).

### Ethics approval and consent to participate

The DTI data used for this study comes from a study approved by the *ethics committee of hospital Clinico San Carlos, Madrid*.

## Supplementary Information

Supplementary Information.

## Data Availability

Please refer to the website http://www.fnsneuralsimulator.org or the GitHub repository http://github.com/fnsneuralsimulator for the download of the simulator. Data concerning the benchmarks is available at the following URL: http://github.com/fnsneuralsimulator/FNS-benchmarks.
