# Supplementary material for: FNS allows efficient event-driven spiking neural network simulations based on a neuron model supporting spike latency
Source: Sci Rep. 2021 Jun 9;11:12160. doi: 10.1038/s41598-021-91513-8 (PMC8190312; doi:10.1038/s41598-021-91513-8)
Supplement: Supplementary file 1 — Supplementary Information. [file 41598_2021_91513_MOESM1_ESM.pdf]

FNS allows efficient event-driven spiking neural network simulations based on a neuron model supporting spike latency

Gianluca Susi[1,2,3*], Pilar Garcés[1], Emanuele Paracone[3], Alessandro Cristini[3], Mario Salerno[3], Fernando Maestú[1,2,4], and Ernesto Pereda[1,5]

[1]Laboratory of Cognitive and Computational Neuroscience (Center for Biomedical Technology), Technical University of Madrid & Complutense University of Madrid, Madrid, Spain
[2]Department of Experimental Psychology, Cognitive Processes and Logopedy, Complutense University of Madrid, Madrid, Spain
[3]School of Engineering, University of Rome 'Tor Vergata', Rome, Italy
[4]Networking Research Center on Bioengineering, Biomaterials and Nanomedicine (CIBER-BBN), Madrid, Spain
[5]Department of Industrial Engineering & IUNE, University of La Laguna, San Cristóbal de La Laguna, Spain
[*]gianluca.susi@ctb.upm.es

SUPPLEMENTARY INFORMATION

Appendix A: LIFL Features

LIFL neuron supports natively the following neurophysiological properties: *integrator*, *spike latency*, *tonic spiking* and *class 1 excitability*. Among these:

- *Tonic Spiking* takes place when a neuron fires a continuous spike train when stimulated through a DC current input[1]. We show this property stimulating a single neuron with a constant spike train (i.e., a discretized DC current input) with amplitude A_c. The raster plot of the spiking neuron activity (i.e., the output neuron response) is reported in Fig. S1. Note that the firing frequency of the neuron is constant.

- *Class 1 Excitability*, exhibited by some cortical neurons, allows the neuron to spike with a frequency that depends on the input strength (ranging from 2 Hz to 200 Hz or more), including the fire at low-frequency when the input is weak. This property allows neurons to encode the input strength into their firing rate[1]. In Fig. S1, we show this behavior stimulating a neuron with a ramp input. Of course, in this case the firing frequency of the neuron is not constant.

In order to further improve the realism of the LIFL neuron, some adjustments can be made at the programming level (as already done for the *refractory period* and the *tonic bursting*), obtaining other computational features.

With regards to the underthreshold decay, FNS gives the possibility to choose among:

- *Linear decay*, assuming $T_l = D\Delta t$ where Δt represents the temporal distance between a couple of consecutive incoming spikes ($D \geq 0$; for $D = 0$ no decay is applied in passive mode, and the neuron behaves as a *perfect integrator*). The

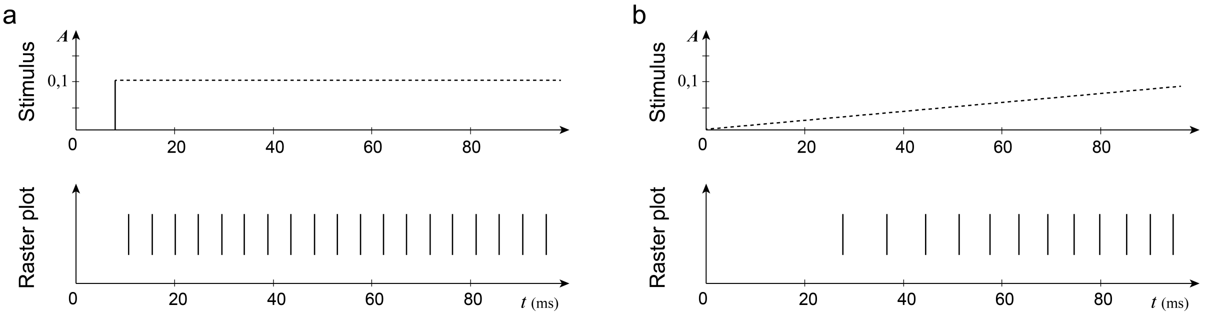

Figure S1. (a): *Tonic Spiking* . Top: *DC* input stimulus with $A_c = 0.1$. Bottom: raster plot (firing activity) of the stimulated neuron, with respect to (simulated) biological time.(b): *Class 1 Excitability* . Top: *ramp* input stimulus with slope $= 0.001$. Bottom: raster plot (firing activity) of the stimulated neuron, with respect to (simulated) biological time. Note that, in order to introduce the external inputs to the event-driven system, we used discrete versions of DC and ramp signals, sampled at constant intervals of $dt = 0.1$

overall update equation then becomes:

$$S_j = S_{p\,j} + A_i \cdot W_{i,j} - D_j \cdot \Delta t \tag{S1}$$

- *Exponential decay*, assuming $T_l = S_{p\,j} \cdot (1 - e^{-\Delta t / D_j})$, obtaining for the overall update equation:

$$S_j = A_i \cdot W_{i,j} + S_{p\,j} \cdot e^{-\frac{\Delta t}{D_j}} \tag{S2}$$

D represents here the classic *time constant*.

Appendix B: T_r calculation

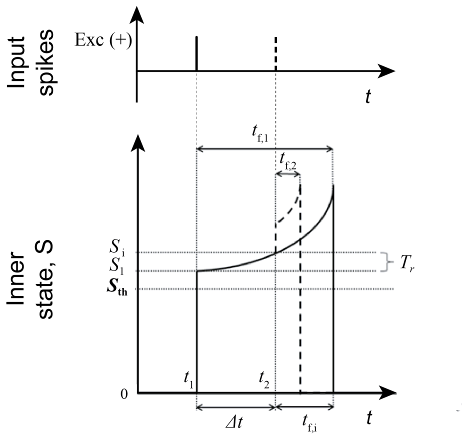

Figure S2. Representation of T_r. LIFL neuron in active mode is characterized by a spontaneous growth of *S*. If a pulse arrives before the actual spike generation, *S* is modified and the t_f will be recalculated. The recalculation considers the intermediate state S_i, i.e., the neuron state at the time the pulse arrives.

Referring to Fig. S2, at the time the neurons inner state is altered from a second input (here excitatory, but non influencial to calculation purposes), the *intermediate state* S_i is determined, and then T_r is calculated.

In event-driven, network elements are updated only when they receive or emit a spike. Once an input spike arrives in active mode, the S_i is calculated on the basis of the time remaining to the spike generation.

Referring to the generic inner state S_i the firing equation is:

$$t_{f,i} = \frac{a}{S_i - 1} - b \tag{S3}$$

We define:

$$\Delta t = t_2 - t_1 \tag{S4}$$

where t_1 and t_2 represent the arrival instants of the synaptic pulses to the considered neuron. Then:

$$t_{f,i} = t_{f,1} - \Delta t \tag{S5}$$

Rearranging Eq. S3, we obtain:

$$S_i = \frac{a}{t_{f,i} + b} + 1 \tag{S6}$$

Now we combine Eq. S5 with Eq. S6

$$S_i = \frac{a}{t_{f,1} - \Delta t + b} + 1 \tag{S7}$$

By defining

$$T_r = S_i - S_1 \tag{S8}$$

where

$$S_1 = \frac{a}{(t_{f,1} + b)} + 1 \tag{S9}$$

and putting Eq .S7 and S9 in S8, we obtain:

$$T_r = \frac{a}{t_{f,1} - \Delta t + b} - \frac{a}{t_{f,1} + b} \tag{S10}$$

that can be rearranged as

$$T_r = \frac{a\Delta t}{(t_{f,1} + b - \Delta t)(t_{f,1} + b)} \tag{S11}$$

Note that we are interested in determining an intermediate state; this implies that we consider the second synaptic pulse only if its timing (i.e., t_2) falls before the spike occurs. This gives us:

$$\Delta t < t_{f,1} \tag{S12}$$

thus we do not have restrictions from the denominator of S11.

The relation S11 can be generalized to the case as more input modify the firing time; then, we can write

$$T_r = S_{ic} - S_{ip} = \frac{a\Delta t_i}{(t_{f,ip} + b - \Delta t_i)(t_{f,ip} + b)} \tag{S13}$$

with

$$\Delta t_i = t_{ic} - t_{ip} \tag{S14}$$

where the subscript ip stays for *intermediate-previous* and ic for *intermediate-current*.

We can also make explicit the dependence of T_r from the previous state, by inverting $t_{f,ip}$ trough Eq. S3, obtaining:

$$T_r = \frac{(S_{ip} - 1)^2 \Delta t}{a - (S_{ip} - 1)\Delta t} \tag{S15}$$

Obviously, the same considerations on the arrival time of the second pulse remain valid, thus we do not have restrictions imposed by the denominator of S15.

Appendix C: An overview of the distributions and functions used in FNS

Distribution of weights

In FNS Gaussian distributions can be used both for the initialization of intra-node weights (one set for excitatory and one set for inhibitory) and inter-node weights of each edge:

$$f(W) = \frac{1}{\sqrt{2\pi\sigma_W{}^2}} exp \frac{(W-\mu_W)^2}{-2\sigma_W{}^2} \tag{S16}$$

where μ_W is the mean, and σ_W^2 is the variance of the distribution. In the formula, W is intended to represent A (distribution of intra-node weights) or ω (distribution of internode weights).

Distribution of edge lenghts

In time, a gamma distribution is implemented for inter-node lengths, which reflects on a gamma distribution of delays. This is characterized by parameters μ_λ (i.e., *mean parameter*) and α_λ (i.e., *shape parameter*). If we call λ the (axonal) delay, the probability density function of a gamma distribution can be written as:

$$f(\lambda) = \lambda^{\alpha_\lambda - 1} \frac{exp(-\lambda\alpha_\lambda/\mu_\lambda)}{(\mu_\lambda/\alpha_\lambda)^{\alpha_\lambda}\Gamma(\alpha_\lambda)} \tag{S17}$$

Note that μ_λ can be defined as:

$$\mu_\lambda = \alpha_\lambda \theta \tag{S18}$$

where θ is known as the *scale parameter*.

Note that with the parameter α_λ is possible to control the type of distribution (low α_λ values lead toward the exponential distribution; high α_λ values lead toward the Dirac distribution); the more α_λ is high, the more the distribution *mode* approaches μ_λ (from the left).

STDP

Synaptic plasticity consists of an unsupervised spike-based process able to modify weights on the basis of the network activity. Considering a synapse connecting two neurons, such mechanism is based on the precise timings of *pre-synaptic pulse* (i.e., the *synaptic pulse* arriving from the pre-synaptic neuron) and *post-synaptic pulse* (i.e., the *output pulse* generated by the post-synaptic neuron), influencing the magnitude and direction of change of the synaptic weight. According to this, in FNS the weight is increased or decreased depending on the pulse order (*pre*-before *post*-, or *post*- before *pre*-, respectively).

In case of inter-node connection the pre-synaptic pulse is taken after the axonal delay block and not before, in order to not to alter information on causality between pulse arrival and pulse generation.

The original STDP behaviour[2] can be approximated by two exponential functions[3].

$$\Delta W = \begin{cases} A_+ \mathrm{e}^{-\frac{\Delta T}{\tau_+}}, \text{ for } \Delta T > 0 & \text{(S19a)} \\ 0, \text{ for } \Delta T = 0 & \text{(S19b)} \\ A_- \mathrm{e}^{\frac{\Delta T}{\tau_-}}, \text{ for } \Delta T < 0 & \text{(S19c)} \end{cases}$$

where ΔT is the difference between post-synaptic pulse generation (i.e., t_{post}) and pre-synaptic pulse arrival (i.e., t_{pre}) instants:

$$\Delta T = t_{post} - t_{pre} \tag{S20}$$

as illustrated in Fig. S3; τ_+ and τ_- are positive time constants for *long-term potentiation* (LTP, S19a) and *long-term depression* (LTD, S19c), respectively; A_+ and A_- are chosen in order to keep weight values bounded between minimum and maximum values).

To make the weight change dependent also on the current weight value, *soft bounds*[4] are introduced in FNS, so that $A_+(W_p) = (W_{max} - W_p)\eta_+$ and $A_-(W_p) = W_p\eta_-$, where W_p is the past value of the synaptic weight, W_{max} the upper bound , and

η_+ and η_- are positive learning constants, usually in the order of $\sim 10^{-5}$. Therefore, the weight update relations implemented in FNS are:

$$W = \begin{cases} W_p + (W_{max} - W_p)\eta_+ \mathrm{e}^{-\frac{\Delta T}{\tau_+}} \text{ , for } \Delta T \geq 0 & \text{(S21a)} \\ W_p - W_p \eta_- \mathrm{e}^{\frac{\Delta T}{\tau_-}} \text{ , for } \Delta T < 0 & \text{(S21b)} \end{cases}$$

where ΔT is the difference between post-synaptic pulse generation (i.e., t_{post}) and pre-synaptic pulse arrival (i.e., t_{pre}) instants; τ_+ and τ_- are positive time constants for *long-term potentiation* (LTP) and *long-term depression* (LTD), respectively; A_+ and A_- are chosen in order to keep weight values bounded between minimum and maximum values.

It is important to stress that the *soft-bounds* approach allows an increase of both the synaptic capacity and the memory lifetime, with respect to the alternative *hard-bounds* approach[5].

In addition, to simplify the STDP event list management, exponential tails are suppressed after a certain time value $TO \cdot max(\tau_+, \tau_-)$, where *TO* is the *STDP timeout constant*, defined by the user, and usually in the order of 100 ms. In this way, spike pairs whose intervals exceed such time limit are not considered for the STDP process.

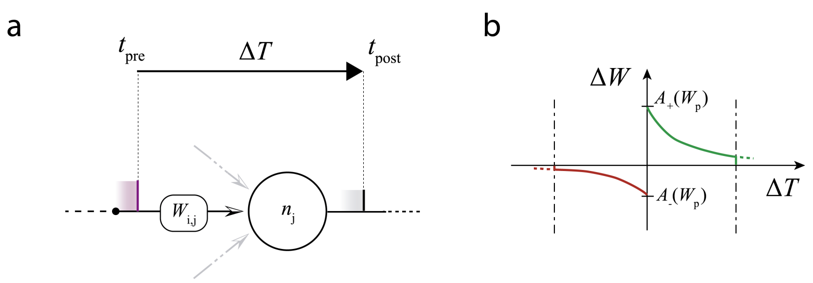

Figure S3. The STDP process in FNS. (a) ΔT calculation in relation to the synapse w_{ij}, considering an inter-module connection (without loss of generality). (b) Shapes of the learning windows (LTP in green, LTD in red) considering exponential tail suppression (dash dot).

Note that values arising from the distribution of inter-node lengths have to be positive to be stored in the internal matrices; in case they assume negative values FNS allows the user to consider the absolute value of such quantities, or to terminate the program execution. In addition, weight values are kept below the value W_{max}.

Appendix D: Data structures

During the initialization phase the program generates the sets of values of weights and delays of the network. Possible values arising from the distributions that are negative or $> W_{max}$ are notified and rectified. Below we briefly describe the main data structures used by the software, highlighting which of these act at the node level:

inter-connection *dictionary*: it is a map containing weight and length of each inter-node connection. The connection is identified by the pre-synaptic neuron (pertaining to node A) and the post-synaptic neuron (pertaining to node B, with $B \neq A$);

intra-connection *dictionary* (node-specific): this is the intra-node equivalent of the *inter-connection* dictionary, where each entry represents the weight of an intra-node connection. The connection is identified by the pre-synaptic neuron and the post-synaptic neuron (pertaining to the same node);

state *dictionary* (node-specific): it contains the inner states of the neurons pertaining to a specific node, and it is constantly updated through the node simulation;

active neuron *list* (node-specific): list of neurons in active mode pertaining to a specific node, sorted on their firing time; this list is constantly updated through the node simulation;

outgoing spike *list* (node-specific): lists of output pulses, including post-synaptic neuron, node and instant, generated from a specific node within a specific time slice;

STDP timing *list*: it temporarily stores event timings in order to compute the ΔW. Such timings are automatically discarded after the *TO* value defined by the user.

Appendix E: Implementation aspects, execution steps and pseudo-code procedure

When a simulation is launched in FNS, two phases are performed in sequence: 1) the *initialization* of the data structures needed by the simulation, and 2) the actual *simulation* of events.

The first phase is carried out through the following steps:

- reading of the *Generator module* and *Neuroanatomical model module* parameters, and the list of NOIs for which the events have to be stored;
- generation of the node-specific data structures and neuron clusters to be run concurrently;
- generation of the global data structures.

After those steps have been accomplished, the second phase begins. The parallelization strategy implemented allows the program to proceed through the sequential simulation of single slices of simulated time with a specific and constant duration, for multiple nodes at the same time. Each cycle terminates with the synchronization between nodes whose events affects each other.

Event-driven procedure

An asynchronous or event-driven algorithm allows the simulation to "jump" from an event to the next one. If the SNN was characterized by identical transmission delays and absence of spike latency, the data structure would be just a *First In First Out* queue, which has fast implementations[6].
At any instant, each network neuron is characterized by its inner state, and active neurons are also characterized by their proper t_f. When a firing event occurs, it propagates toward the target neurons taking into account the connection delays (if present). Such events modify the inner state of post-synaptic neurons (and their t_f, for the active neurons), on the basis of the amplitude and sign of the pulse, and the time elapsed from the last state update. Four different cases of state update can happen to the target neuron:

passive-to-passive. This does not have any effect on the event list

passive-to-active. This elicits the insertion of an event (and related firing time) orderly on the event list

active-to-active (i.e., post-trigger anticipation/postponement). This elicits the update (and reordering) of its firing time on the event list

active-to-passive (i.e., post-trigger inhibition). This elicits the elimination of an event (and related firing time) from the event list

In addition to the four cases listed, two "forbidden" cases can occur during the simulation: from *passive mode* to $S < 0$ and from *active mode* to $S \geq S_{max}$; for such cases, specific actions are included in the procedure (state value correction and output spike production, respectively).

At the same time, weights for which plasticity is active are updated accordingly, taking in account the *STDP timing list* (see *Appendix D*).

Parallelization

In a parallel computing scenario the problem is splitted in many sub-problems such that their solutions can be computed independently and then collected to provide the global solution. In simulations of brain networks it is not trivial to determine which events can be executed in parallel because of the intricate cause-effect relations between the network elements. This led to the development of specific strategies for parallelising event-driven SNNs avoiding causality errors[7–12].
The event-driven parallelization method on which FNS is based on can be defined as *adaptive*[7], since the algorithm chooses an appropriate network-specific interval of simulated time to be used for the synchronization of the parallel tasks, avoiding as much as possible the underuse of available hardware resources. Given a generic network, the *opaque period* (OP) is the minimum simulated time needed by a signal to travel from a network element to an adjacent one[13]. Then, within any simulated time window smaller than the OP of the network, each event cannot be caused by (or cannot affect to) any other event happened during the same time window.

If ev_i and ev_j are two distinct events such as

$$\left|\tau(ev_j) - \tau(ev_i)\right| < OP, \quad i \neq j \tag{S22}$$

then they can be computed in parallel without loss of cause-effect relationship. This allows us to parallelize the computation within the *time slice* $T_s < OP$. On the other hand, each unit must wait until each of the others has ended to simulate the events of the previous T_s to process new information; then, a *sync step* is needed to "deliver" the events just calculated to the unit which should use them to produce new events during the next *OPs*.

In the case of neural computation, an event could affect another one in a very short time, leading to a short *OP*, then counteracting the benefit of the parallelization. In order to efficiently perform parallel computation, in FNS the following strategy is adopted:

- each node is assigned to a specific *thread* (i.e., a process that deals with a local problem);
- the T_s duration is sized as the minimum among all the network inter-node connection delays (that we define *Bounded Opaque Period* (BOP)), since this is the shortest interval needed by a neuron of a node to affect the state of a neuron of another node.
- inter-node spikes in queue are delivered to the corresponding threads through the *synch step*, at intervals equals to the BOP. Once a thread gets a spike event from a node, it puts this event orderly to the internal node-specific *active neuron list* and updates the internal state of the post-synaptic neuron at the proper time.

If the network presents two or more inter-connected nodes with zero-delay, it is possible to make FNS consider these nodes as a single *macro-node*, and the mechanism continues to be valid. The fact of considering the more ample concept of *macro-node* enables the possibility of representing heterogeneous regions without losing the parallelization feature.

Threads are executed in parallel by the *thread workers* (i.e., multi-threaded or hyperthreaded CPUs), each of which can execute at most a thread at a time. In order to minimize the processing times, each worker can serve queued threads in turns for a short time, and different workers can swap threads each other, to achieve a dynamic balancing of the computational load.

The current version of FNS is written in Java ®. The parallelization mechanism introduced, conceived for a multi-threaded scenario with shared memory, benefits from specific features of Java oriented to the optimization of memory resources, as the *Java Garbage Collector*[14]. Below, we report the execution steps, and a description in pseudo-code of the procedure implemented in FNS, supporting the synchronization mechanism between nodes.

Memory usage and periodic dumping

FNS integrates a serialization mechanism by which the simulated data is periodically saved to disk during a run. This function is necessary to face long simulations and obtain the output signals without dealing with memory issues. FNS contemplates a default *serialization buffer* S_b (i.e., the amount of simulation data that FNS keeps in memory before writing to disk), that can be modified by the user to optimize the performance, in terms of simulation times or memory usage. However, S_b should be handled carefully, and its modification may have to be accompanied by that of the *heap size* of Java (i.e., the amount of memory reserved for the storage of Java objects), as reported in *Appendix F*. In the following section these aspects have been taken in account to maximize the simulator performance.

Execution steps and preudo-code procedure

Each *thread* can assume one of the three states *running*, *waiting* or *runnable*. When the whole simulation starts, the steps until the completion of the execution are the following:

1. all threads are set as *runnable*;
2. each of them simulates the generation of events within the (simulated) BOP_1 (i.e., the time window from time $t = 0$ to time $t = \bar{B}$). If empty threads occur, for them the simulation jumps directly to point 3;
3. once a thread has generated all the events within the current BOP_1 window, it sets its status to *waiting*;
4. once all threads have completed to simulate the events of the BOP_1 window, all threads synchronize each other through the synch step;

5. all threads get again to the state *runnable* and simulate the events generated within the BOP_2 (i.e., the time window from time $t = \bar{B}$ to time $t = 2\bar{B}$), and so on.

This algorithm cycles until the stop condition set on the overall simulated time t_{stop}.
Obviously, a firing event generated within BOP_n not necessarily will be delivered as burning event during BOP_{n+1}: it could be delivered in one of the following BOPs, depending on the connection delay involved.
The concept of parallelization through BOP is summarized in Fig. S4, where for simplicity we consider the simple *resonance pair* motif[15,16] (i.e., two nodes bidirectionally connected with delay).

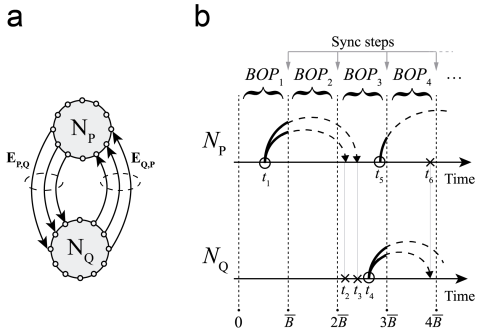

c

SYNC-STEP REFERENCE	INTER-NODE CONNECTION PARAMETERS	TARGET NODE	BURNING TIME
BOP_1	$\omega_{i,j}, \lambda_{i,j}$<br>$\omega_{i,k}, \lambda_{i,k}$	N_Q<br>N_Q	$t_2(BOP_3)$<br>$t_3(BOP_3)$
BOP_2	*null*	*null*	*null*
BOP_3	$\omega_{l,m}, \lambda_{l,m}$<br>$\omega_{l,n}, \lambda_{l,n}$<br>$\omega_{o,p}, \lambda_{o,p}$	N_P<br>N_P<br>N_Q	$t_6(BOP_4)$<br>...<br>...
...	...	...	...

Figure S4. BOP-based parallelization mechanism. (a) Example network: two interconnected nodes are generating spiking activity (under proper external stimulation, not shown in the figure). (b) Temporal diagram of the parallelization process. Inter-node pulses are represented by dashed arrows, firing events by circles, burning events by crosses. An inter-node firing event generates burning events in the target node. The delivery of the events to the burning node will happen at the end of the firing event's BOP. Given a firing event, related burnings will happen in one (or more) following BOPs. Given a burning event, the related firing event belongs to a BOP that precedes the current one (not necessarily the previous one). The axis represents simulated time. (c) Event schedule grid.

Pseudo-code procedure

· let `spike_queue` be the list of all spikes generated by any pre-synaptic neuron sorted in ascending order of spike time (the first spike in the list is the one with the least spike time);

· let `outer_burning_event`: the list of spikes to be delivered to post-synaptic neurons belonging to outer nodes. At each synchronization:
1. each entry of this list is read and sent to the thread executing the routine for the right node;
2. after each item in the list has been sent, the whole list is cleared.

· let `current_time` be the current simulated time;

· let `split_stop_time` be the stop time for the current BOP simulation;

· let `final_stop_time` be the simulated time at which the whole simulation must stop;

· let `run_burning_routine(s*)` be the routine which calculates all the burning events caused by the fire event hold by the spike `s*`: during this procedure, all the burning events involving post-synaptic neurons of outer nodes are stored to a special `outer_burning_event`;

· let `send_fires_for_outer_nodes()` be the routine which sends the spikes stored in `outer_burning_event` to post-synaptic neurons in outer nodes;

· let `update_incoming_spikes_queue()` be the routine which updates the `spike_queue` with the spikes coming from outer nodes and targeting post-synaptic neurons of the present node before beginning the next BOP simulation.

```
while true:
   while current_time < split_stop_time:
      s* = spike_queue.pop()
```

```
      run_burning_routine(s*)
      if current_time >= split_stop_time:                        (1)
         if split_stop_time >= final_stop_time:                  (2)
            return
         send_fires_for_outer_nodes()
      wait_until(current_time < split_stop_time)                 (3)
   update_spikes_queue()
```

```
(1) end of the BOP: send the fires to the outer nodes
(2) end of the last BOP: end of the node simulation
(3) stops the thread until split_stop_time is updated
    with the next BOP stop time
```

Appendix F: Performance optimization and simulation details

Performance optimization

To optimize the performance of FNS, we can act on the parameter S_b and the Java *heap size*. This can be done by considering two aspects:

- the memory used by FNS can be decreased by reducing the heap size (both minimum and maximum allocated heap size can be modified in Java). But note that the heap can be reduced down to a critical value (a further decrease would result in longer simulation times and/or the generation of out-of-memory errors);
- On the other hand, for a given value of maximum heap size, a greater size of S_b (i.e., a smaller *dumping frequency*) would allow the threads to work with more continuity, thus reducing simulation times. Especially in a parallel scenario, frequent file writing generates a behavior that can be deleterious for simulation times. But note that the S_b can be increased up to a critical point (further increase of S_b would result in longer simulation times and/or the generation of out-of-memory errors due to the boundary imposed by the heap size).

Complementary information on the benchmarks

For the simulations we used FNS v.3.3.92 and NEST v.2.20. As for NEST, during the simulations we activated the recording of spike times and membrane potential for each network neuron, using the NEST recording devices *spike_detector* and *multimeter*, respectively (options *"to_file":True*, *"to_memory": False*, and *"time interval"* set to the *dt* used for the simulation). In addition, we enabled the thread-parallelism to speed up the simulation (options *"local_num_threads": 8* as the number of threads of the machine used for the simulations.

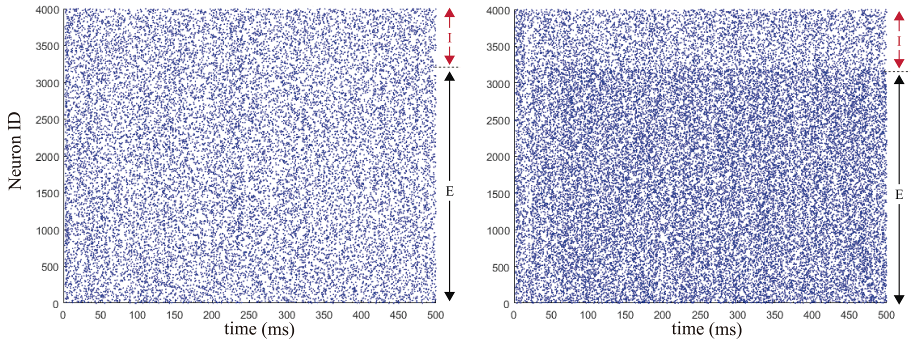

Figure S5. Rasterplots from the two benchmarks. Spiking activity obtained in FNS from the node of 4000 neurons of the benchmark A (left), and one of the 14 nodes of the benchmark B (right). In the latter case, the mean firing rate is visibly increased due to the weighted interconnection among the excitatory neurons (E, which ID range is indicate with the black arrow on the right) of the network nodes.

Analogously, for FNS we selected the reduced format for the output file (obtained with the switch $-r$ after the simulation command), which activates the recording of the spike times and internal state of the network neurons, avoiding the recording of

other simulation variables as synaptic weights. With regards to the resolution, we have not limited the original precision of FNS, which uses floating-point variables for both times and internal states.

In order to generate the "skeleton" of the network of the benchmark B, we considered the 14 regions composing the *DMN*, a *task-negative* resting state network, which is more strongly active during idling states than during task performance. Using the DTI data of real subjects[17], we initialized the number of connections of each network edge, N_e, in proportion to the average number of streamlines detected between the brain regions composing the DMN (for the method see the reference study), while for the lengths associated to the network edges, μ_λ, we have considered the euclidean distances between the centroids of the brain regions. In order to obtain the delay values for each edge, we considered an axonal speed of $5.1m/s$. Connections between nodes are limited to act between excitatory pools as they are here considered to represent long-range axons of pyramidal neurons, as in previous computational neuroscience studies[18]. The final structural model counts 112k neurons (56k network neurons and 56k external spike generators) and 5140k synapses (of which 4480k intra- node connections, $\sim 100k$ inter-node connections, and 560k input connections).

Table 1. Summary of the LIFL model parameters used in the benchmarks A and B

D_{exc} (exp.decay)	D_{inh} (exp.decay)	t_{arp}	N_b	A_{exc}	A_{inh}	$\mu_{w,exc}$	$\mu_{w,inh}$
20	20	5	0	0.025	0.225	1	1

In Fig. S5, the spiking activity obtained in FNS from the node of 4000 neurons of the first benchmark is compared with the activity of one of the nodes of the second benchmark. As for FNS we used in both the benchmarks the LIFL model with exponential decay, which set of parameters is reported in 1.

Table 2. Recommended values of *heap size* for benchmarks A and B

.

**Network ID**	**Recommended heap size**
A1	$\sim 200m$
A2	$\sim 300m$
A3	$\sim 400m$
B	$\sim 1600m - 2000m$

A S_b of value 100 has been used for all the simulations. Following the observations above regarding the memory usage, we carried out the simulations using the *heap size* values summarized in the table 2. Note that a higher heap value can determine a higher RAM allocation, but it does not always lead to an improvement in the simulation performance. Instructions on how to change the Java heap size are available on the FNS website and on the official Java website.
The experiment packages to reproduce these simulations are available on the github page of FNS (at the following URL: `github.com/fnsneuralsimulator/FNS-benchmarks`).

Appendix G: Approximating the alpha function using *RDI*

With the RDI approach we can characterize the conductance's rise and decay with different functions (i.e., linear, or exponential, or a combination of both), which we choose depending on the problem we are facing and the computational effort we can afford. To show this mechanism, we start defining the alpha function:

$$\alpha(t) = u(t) \cdot S_{peak} \cdot \frac{t}{\Delta t_{peak}} \cdot e^{1-\frac{t}{\Delta t_{peak}}} \tag{S23}$$

where $u(t)$ is the Heaviside function, S_{peak} is the maximum value of the alpha function and Δt_{peak} the time necessary to achieve S_{peak}.
To mimick this curve, we will generate a piecewise-defined function which *change-point* (i.e., the point of the curve which demarcates rise and decay phases) coincides with the maximum of $\alpha(t)$. The Δt_{peak} can be modulated by the amplitude of the input spikes, or it can be chosen constant, but we will examine only this second case for simplicity. Let's consider a spike sent by neuron n_i, arriving at the time $t = 0$ to neuron n_j, which was at the symbolic resting potential $S_j = 0$. We can now analyze the two approximations:

- *Linear-linear approximation*. For the rise phase, we just need to set the slope of the line passing through the points $(0,0)$ and $(\Delta t_{peak}, A_i W_{i,j})$. The decay phase is also easy to characterize since we know the presumed change-point, i.e. $(\Delta t_{peak}$, $A_i W_{i,j})$, and the linear decay constant.

- *Exponential-exponential approximation*. The decay phase is easy to characterize since we know the presumed change-point, i.e. $(\Delta t_{peak}$, $A_i W_{i,j})$, and the exponential decay constant. For the rise phase, the family of rising exponential (increasing and concave-down) functions that intersects the change-point are characterized by the following relation:

$$\tau_{rise} = -\frac{\Delta t_{peak}}{log(\frac{-S_{peak}}{k} + 1)} \tag{S24}$$

  where $k(> S_{peak})$ is the *curvature constant* of the rising exponential. Finally, to scale the rise curve depending on the amplitude of the input spike, a simple choice would be to set a fixed value for τ_{rise}, and multiply it by a constant to obtain a vertical elongation.

Obviously, we can introduce some variants, e.g. if we want the rise constants to be modulated by the current internal state (at the expense of a greater computational effort).
Note that the RDI approach allows the user to set the two functions independently, or even mix different functions (*linear-exponential*, or *exponential-linear*), making this synapse model even more versatile than the alpha function, where the time courses of the rise and decay are correlated.
To introduce this effect in our system, we simply add two steps to the original algorithm, such that, for each incoming spike to the target neuron n_j:

1. Δt_{peak} is used to temporally shift the start of the decay phase or latency period (i.e., $t_{arrival} + \Delta t_{peak}$). This variable allows us to define the duration of the rise phase, as well as to take track of possible new arrivals during this interval and, in that case, to recompute S_{peak}. Note that, although $A_i \cdot W_{i,j}$ is not used instantaneously, is central to know if the current contribution will generate a mode transition in the target neuron (i.e., *passive-to-active*, see *Appendix E*).

2. When compute the time difference with the previous arrival to the same target neuron, check if the current spike falls in a rise period, i.e., between $t_{arrival,p}$ and $t_{arrival,p} + \Delta t_{peak,p}$.

- If yes, recalculate the new expected maximum S_{peak} and update the Δt_{peak}, taking in account $S_{peak,p}$ in order not to repress the residual increase from the previous contribution.

- If not, simply compute the new S_{peak} considering the residual S in the underthreshold phase, considering that it has started at time $t_{arrival,p} + \Delta t_{peak,p}$

It is important to rember that the event driven strategy allows us to calculate isolated points of the piece-wise function, i.e., only in case a new spike is received.
Please note that this function is not implemented in the current version of FNS and will be added in future releases.

References

1. Izhikevich, E. M. Which model to use for cortical spiking neurons? *IEEE Transaction on Neural Networks* **15**, 1063–1070 (2004).

2. Bi, G. & Poo, M. Synaptic modifications in cultured hippocampal neurons: dependence on spike timing, synaptic strength, and postsynaptic cell type. *The J. Neurosci.* **18**, 10464–10472 (1998).

3. Abbott, L. F. & Nelson, S. B. Synaptic plasticity: taming the beast. *Nat. Neurosci.* **3**, 1178–1183 (2000).

4. Sjöström, J. & Gerstner, W. Spike-timing dependent plasticity. http://www.scholarpedia.org/article/Spike-timing_dependent_plasticity (2010).

5. van Rossum, M. C., Shippi, M. & Barrett, A. B. Soft-bound synaptic plasticity increases storage capacity. *PLOS Comput. Biol.* **8**, 1–11 (2012).

6. Cormen, T., Leiserson, C., Rivest, R. & Stein, C. *Introduction to algorithms, 2nd ed.* (The MIT Press, 2001).

7. D'Haene, M. A framework for parallel event driven simulation of large spiking neural networks. In *7e FirW Doctoraatssymposium* (2006).

8. Mouraud, A. & Puzenat, D. Simulation of large spiking neural networks on distributed architectures. the “DAMNED” simulator. In Palmer-Brown, D., Draganova, C., Pimenidis, E. & Mouratidis, H. (eds.) *Engineering Applications of Neural Networks.*, 359–370 (Springer International Publishing, 2009).

9. Lobb, C., Chao, Z., Fujimoto, RM & Potter, S. Parallel event-driven neural network simulations using the hodgkin-huxley neuron model. In *Principles of Advanced and Distributed Simulation (PADS)*, 16–25, DOI: 10.1109/PADS.2005.18 (2005).

10. Grassmann, C. & Anlauf, J. Distributed, event driven simulation of spiking neural networks. In *Proceedings of the International ICSC - IFAC Symposium on Neural Computation*, 100–105 (1998).

11. Djurfeldt, M. *et al.* Massively parallel simulation of brain-scale neural network models. Tech. Rep., “KTH” School of Computer Science and Communication, Stockholm, Sweden (2005).

12. Delorme, A. & Thorpe, S. SpikeNET: an event-driven simulation package for modelling large networks of spiking neurons. *Netw. Comput. Neural Syst.* **14**, 613–627 (2003).

13. Lubachevsky, B. Efficient distributed event-driven simulations of multiple-loop networks. *Commun. ACM* **32**, 111–123 (1989).

14. Pufek, Grgic & Mihaljevic. Analysis of garbage collection algorithms and memory management in java. In *2019 42nd International Convention on Information and Communication Technology, Electronics and Microelectronics (MIPRO)*, 2623–8764 (IEEE, Opatija, Croatia, 2019).

15. Gollo, L., Mirasso, C., Sporns, O. & Breakspear, M. Mechanisms of zero-lag synchronization in cortical motifs. *PLOS computational biology* **10**, 1–17, DOI: 10.1371/journal.pcbi.1003548 (2014).

16. Maslennikov, O. V. & Nekorkin, V. I. Modular networks with delayed coupling: Synchronization and frequency control. *Phys. Rev. E* **90**, 012901, DOI: 10.1103/PhysRevE.90.012901 (2014).

17. Garces, P. *et al.* The Default Mode Network is functionally and structurally disrupted in amnestic mild cognitive impairment - a bimodal MEG DTI study. *Neuroimage Clin.* **6**, 214–221 (2014).

18. Nakagawa, T. *et al.* How delays matter in an oscillatory whole-brain spiking-neuron network model for MEG alpha-rhythms at rest. *Neuroimage* **87**, 383–394 (2014).
